# Knowledge, protective behaviours, and perception of Lyme disease in an area of emerging risk: results from a cross-sectional survey of adults in Ottawa, Ontario

**DOI:** 10.1186/s12889-024-18348-6

**Published:** 2024-03-20

**Authors:** James J. Logan, Michael Sawada, Anders Knudby, Tim Ramsay, Justine I. Blanford, Nicholas H. Ogden, Manisha A. Kulkarni

**Affiliations:** 1 School of Epidemiology and Public Health, University of Ottawa, Ottawa, ON Canada; 2 Laboratory for Applied Geomatics and GIS Science (LAGGISS), Department of Geography, Environment & Geomatics, University of Ottawa, Ottawa, ON Canada; 3 Department of Geography, Environment & Geomatics, University of Ottawa, Ottawa, ON Canada; 4 Ottawa Hospital Research Institute, University of Ottawa, Ottawa, ON Canada; 5 Department of Earth Observation Science, Faculty of Geo-Information Science and Earth Observation, University of Twente, Enschede, Netherlands; 6 Public Health Risk Sciences Division, National Microbiology Laboratory, Public Health Agency of Canada, Saint-Hyacinthe, QC Canada

**Keywords:** Lyme disease, Prevention, Protective behaviours, Risk factors, Ticks, Tick bites, Tick-borne diseases, Health knowledge, Attitudes and practices

## Abstract

**Background:**

The number of Lyme disease risk areas in Canada is growing. In regions with emerging tick populations, it is important to emphasize peridomestic risk and the importance of protective behaviours in local public health communication. This study aims to identify characteristics associated with high levels of Lyme disease knowledge and adoption of protective behaviours among residents in the Ottawa, Ontario region.

**Methods:**

A geographically stratified web survey was conducted in November 2020 (*n* = 2018) to determine knowledge, attitudes, and practices regarding Lyme disease among adult residents. Responses were used to calculate: (i) composite scores for knowledge and adoption of protective practices; and (ii) an exposure risk index based on reported activity in woodlands during the spring-to-fall tick exposure risk period.

**Results:**

60% of respondents had a high knowledge of Lyme disease, yet only 14% indicated they often use five or more measures to protect themselves. Factors strongly associated with a high level of Lyme disease knowledge included being 55 or older (Odds Ratio (OR) = 2.04), living on a property with a yard (OR = 3.22), having a high exposure index (OR = 1.59), and knowing someone previously infected with Lyme disease (OR = 2.05). Strong associations with the adoption of a high number of protective behaviours were observed with membership in a non-Indigenous racialized group (OR = 1.70), living on a property with a yard (OR = 2.37), previous infection with Lyme disease (OR = 2.13), prior tick bite exposure (OR = 1.62), and primarily occupational activity in wooded areas (OR = 2.31).

**Conclusions:**

This study highlights the dynamics between Lyme disease knowledge, patterns of exposure risk awareness, and vigilance of personal protection in a Canadian region with emerging Lyme disease risk. Notably, this study identified gaps between perceived local risk and protective behaviours, presenting opportunities for targeted enhanced communication efforts in areas of Lyme disease emergence.

**Supplementary Information:**

The online version contains supplementary material available at 10.1186/s12889-024-18348-6.

## Background

Lyme disease is a tick-borne bacterial illness caused by *Borrelia burgdorferi* transmitted, in the North American northeast, through the bite of infected *Ixodes scapularis* (blacklegged) ticks [1]. It is the most common vector-borne disease affecting humans in North America and Europe [2, 3]. Since Lyme disease became nationally notifiable in Canada, the annual number of reported cases has risen dramatically from 144 in 2009 to 3,147 in 2021 [4]. In Ottawa, Ontario, only six human cases were reported in 2008 compared to 190 in 2017, when public health officials first declared the region an at-risk area [5, 6]. Recent studies in this region demonstrate that reproducing blacklegged tick populations are becoming established and that nearly 30% of ticks tested carry *B. burgdorferi* [7, 8]. This rapid rise in human cases and concern for other emerging diseases highlights the importance of surveillance activities and efforts to improve public awareness in areas of emerging tick-borne disease risk [9, 10].

Risk factors for Lyme disease are primarily related to individual behaviours involving tick exposure in areas with suitable tick habitat, such as woodlands, gardens, residential lawns, and public greenspaces [11]. In locations where blacklegged tick populations are becoming established, peridomestic risk is a legitimate and growing concern [12]. There is evidence that public health interventions applied at the neighbourhood scale, comprised mainly of strategies aimed at reducing local entomological hazard, are likely effective [11]. However, even within such high-risk areas, the degree of correlation between tick abundance and human Lyme disease incidence at the level of residential properties is not well established [13]. Thus, tick control interventions cannot exclusively minimize risk, which highlights the need for actions taken by individuals to protect themselves. Household and personal protective practices are the most effective form of protection against tick-borne illnesses [14, 15].

Actions that individuals can take to protect against Lyme disease include the application of insect repellents, wearing long clothing that covers the legs, tucking pants into socks, and performing tick checks after activity in areas with woodlands or long grass [15–17]. One review found that the effectiveness of these commonly used measures in reducing tick-borne disease rates was inconclusive and highlighted the insufficient collection of details regarding how these measures are practiced (e.g., use of one measure at the expense of others, selective combinations of measures) as a potential explanation [14]. Studies in areas where tick-borne diseases are endemic demonstrate that local Lyme disease incidence rates and perceived level of personal risk significantly influence the adoption of measures to prevent exposure and infection [18].

National awareness of Lyme disease has risen over time in Canada, but routine adoption of preventive practices is not high among Canadians [19, 20]. While increased awareness is encouraging, the gap between awareness and the attitudes and behaviours contributing to the practice of personal protection raises concerns. The recent and ongoing expansion of suitable habitat for blacklegged ticks in Canada means that individual regions and, more directly, municipalities are experiencing growing risk [8, 21–23]. Even in the eastern United States, where Lyme disease is highly endemic, calls for local and environmental prevention measures have increased as the tick hazard has become more geographically widespread [15, 24]. However, some evidence suggests that programs that plan and implement landscape controls to reduce tick populations are insufficient as they cannot uniformly reduce the risk by simply reducing the hazard [12, 15]. This is because differences amongst people in the frequency of their interactions with tick habitat and use of protective behaviours contribute to variability in personal risk. It is therefore essential to identify characteristics of individuals associated with high exposure potential to target public health education campaigns that encourage the regular use of multiple protective measures.

As a model of disease prevention, Protection Motivation Theory (PMT) proposes that behavioural choices are a response to an individual’s perception of the severity and their own vulnerability to disease, as well as whether they can reduce their own risk by adopting recommended actions [25]. Applied to Lyme disease prevention, PMT posits that people’s motivation to adopt personal protective behaviours relies on them (1) perceiving Lyme to be a serious illness, (2) believing they have an elevated risk of exposure to ticks, and (3) trusting that the recommended action(s) will be effective in preventing Lyme disease. A Scandinavian study applying PMT to tick bite protection found that participants’ belief in the seriousness of tick bites and Lyme disease predicted the adoption of protective measures, but that the perceived likelihood of exposure was associated with little change in the measures used [26].

As of 2017, 89% of adults in Ottawa were aware of Lyme disease [5]. Of those adults, just over 80% knew that Lyme disease results from tick bites and 62% reported using at least one protective measure to avoid exposure [5]. These rates are slightly lower than province-wide estimates reported in a 2014 national survey [19]. Though this level of Lyme disease awareness remains consistent at the Canadian level in 2023, little improvement regarding the adoption of behaviours aimed at tick bite prevention has occurred nationally [27]. A 2012 study evaluating the knowledge, attitudes, and practices for preventing Lyme disease held by the population of the Montérégie region of Québec found the epidemiological status of a region and behaviours of population subgroups were associated with the adoption of individual protective behaviours [18, 28]. More recently, results from a survey of the neighbouring Estrie region in Québec demonstrated low adoption of preventive behaviours against tick bites with high sub-regional variability in this area of Canada experiencing high Lyme disease incidence [29, 30]. Over the past five years, the establishment of the tick vector across the Ottawa municipal region has continued to increase and, though most Lyme disease cases reported to Ottawa Public Health suspect their tick encounter occurred during travel outside of the city, the characterization of peridomestic Lyme disease incidence in Ottawa neighbourhoods hints at localized hazard in the community [7, 8, 10, 31].

The main objective of this study was to evaluate the level of Lyme disease knowledge and adoption of preventive practices in a Canadian municipality with emerging Lyme disease risk, namely Ottawa, Ontario. We further aimed to compare these observations across residents of five city regions (i.e., urban, rural, and three suburban areas defined in the city’s east, south and west) and population subgroups, as well as to identify individual characteristics associated with high levels of Lyme disease knowledge and adoption of protective behaviours.

## Methods

### Study design

**Fig. 1:**
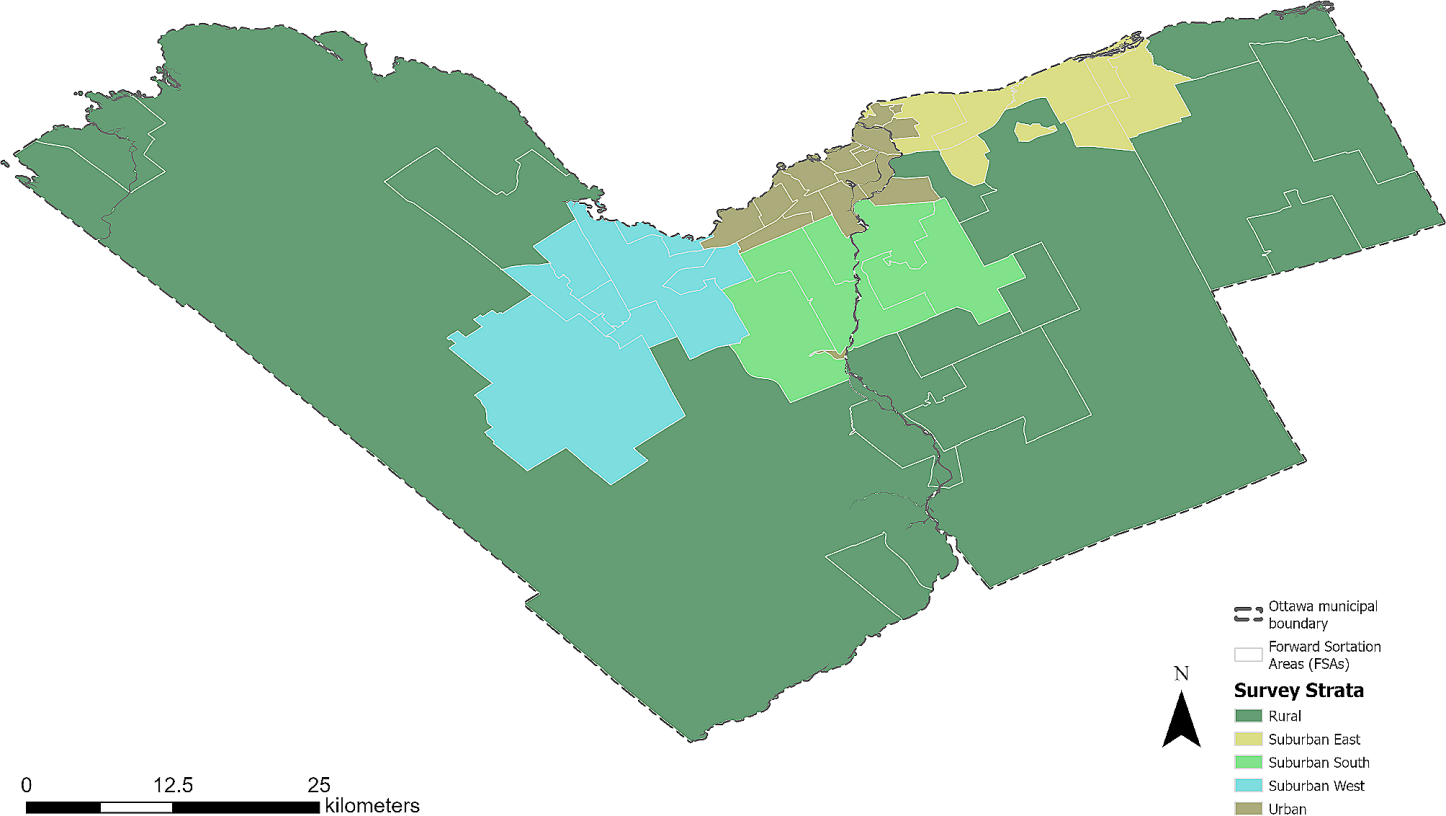
The geographic extent of survey strata defined by Canadian Forward Sortation Area of home address

This cross-sectional study evaluated data collected through a web survey of Ottawa-area adults in November 2020. To be eligible, participants had to respond in either English or French. Canadian survey and analytics company Leger randomly invited participants from web panels of recruited Ottawa residents from November 5 to 25, 2020 [32]. Leger applied recruitment quotas to achieve a sample representative of the Canadian population. We used a stratified approach to ensure a balanced geographic representation across the city of Ottawa (i.e., urban, rural, suburban west, suburban south, and suburban east). We defined these five strata using statistical estimates of population density, walkability, and car commuting rates calculated by the Ottawa Neighbourhood Study (ONS) [33]. Following Statistics Canada definitions, we used population density to identify natural neighbourhoods of the ONS as either urban (greater than 400 people per square kilometre) or rural (less than 400 people per square kilometre) [34]. Following guidelines for categorizing suburbs established elsewhere [35] we sub-selected natural neighbourhoods where car commuter rates were above the City of Ottawa average (69%) or where ONS walkability measures implied moderate car dependence (i.e., scores between 20 and 50) as suburban. Using the Ottawa Greenbelt, an expanse of protected green space that stretches from east to west around the city, as a natural boundary, we then geographically subdivided the suburban neighbourhoods into areas east, south, or west of the urban core. Leger recruited participants using the Forward Sortation Area (FSA) of their home addresses, using the FSAs to apply an approximation of these neighbourhood-based strata (Fig. 1, Supplementary file 1). We aimed to obtain 370 responses from individuals aged 18 or older per stratum. This sample size would allow us to estimate, at a 95% confidence level and with a 5% margin of error, whether the proportion of Ottawa respondents with a high level of knowledge concerning Lyme disease was significantly different from 60% (a proportion that is slightly greater than that reported for the entire province of Ontario in a previous national survey [19]).

### Survey instrument

We used a survey instrument designed for this study, adapted from questions used in a national study that evaluated Lyme disease knowledge, attitudes and practices of the Canadian population, as well as the Ottawa Public Health Rapid Risk Factor Surveillance survey for Lyme disease [20, 36]. The questionnaire (see Supplementary file 2) included questions assessing respondents’ knowledge concerning Lyme disease (e.g., its transmission, means of protection, symptoms, and treatment), perception of local risk, personal attitude towards possible Lyme disease prevention measures, personal adoption level of preventive behaviours (i.e., “In Ottawa I apply this measure to protect myself from Lyme disease:” always; frequently; rarely; never; sometimes I apply this measure, but not to protect myself from Lyme disease; does not apply to my situation; I prefer not to answer), as well as experience with tick bites and Lyme disease. We also asked respondents how frequently they visited “woodlands or areas with tall grass and/or shrubs” during the Lyme disease risk season from May to October of the survey year. To supplement this information on self-estimated exposure risk, we used an interactive map where respondents could identify up to three locations in “wooded areas or areas with tall grass and/or shrubs” as sites that they frequently visited over the same period and asked respondents to identify the primary reasons for such visits (e.g., work, recreation, camping). We also collected information on respondent demographics (age, gender, ethnicity, household income, and education level) to evaluate population subgroups. In analyses, we categorized respondents as White, Indigenous, or other based on their responses and guided by the Ontario Human Rights Commission’s definitions of belonging to racialized population groups [37].

### Data analysis

#### Lyme disease knowledge and personal practices scores

We used composite scores to identify high levels of Lyme disease knowledge and adoption of personal practices. To obtain Knowledge Scores (KS) ranging from 0 to 4, we totalled the scores from four knowledge questions, including those on the transmission of Lyme disease to humans (i.e., “by a tick bite”), tick removal strategies (i.e., “pull it out with tweezers/forceps/etc.”), the first symptom of Lyme disease in humans (i.e., any or all of “headache,” “fever,” “reddish rash on the skin,” and “lethargy/malaise”), and existing treatment strategies (i.e., “with antibiotics in tablet form”). We assigned one (1) point for each question where only the correct option(s) were selected. For questions where participants selected ‘other’ as part of their response, we re-categorized the answer as correct if it was the only additional selection and the free response provided was an appropriate expansion of the expected answer (e.g., “twist it out with tweezers,” “making sure to get the head out”). We similarly calculated personal practices scores (PS) between 0 and 7 based on the sum of scores for self-reported protective measures applied for the purpose of protecting against Lyme disease - one (1) point for each response of “always” or “frequently” using the following: “seeking and removing ticks on yourself after a stay in a forested area,” “wearing long clothes that cover the legs,” “using insect repellants with DEET or Icaridin on skin and/or clothing,” “wearing clothing treated with an insecticide,” “avoiding woodlands during the spring-to-fall risk period,” “putting pesticides on my property,” and “mowing the lawn regularly on my property.” This approach was chosen to reflect regular use of the protective practice in question for the purpose of Lyme disease prevention.

In all analyses, we dichotomized scores to identify individuals with a high level of knowledge regarding Lyme disease and high adoption rate of personal protective practices, allowing comparability with other Canadian Lyme disease studies [18–20, 38]. According to the common practice of using Bloom’s cutoff points, a good score is considered 80–100% of possible answers scored as correct [39, 40]. With total possible scores for KS and PS of 4 and 7, respectively, we defined our threshold for “High” scores with a modified Bloom’s cutoff of 70% [39, 40]. With low totals for each domain and, in the case of protective measures, to allow that respondents may have specified “does not apply to my situation” for some practices, we used the modified cutoff point to remain conservative in what was considered “high”. Thus, a KS of three or four represented an accuracy greater than 70% and was considered high Lyme disease knowledge. We similarly considered PS of 5 or greater to be “high”, representing the personal adoption of more than 70% of the surveyed protective measures.

#### Exposure risk classification

From the specific locations volunteered by respondents as the wooded areas or areas with long grass that they frequented most, we identified whether individuals visited sites with high or low risk of blacklegged tick presence. We classified sites in Ontario with a 35% or higher probability of blacklegged tick presence, according to the ecological niche model developed by Slatculescu et al. [41], as “high” risk. This threshold was selected using the model output statistic aimed at maximizing model sensitivity and specificity - intended as a more conservative estimate of what classifies as a “high-risk” area and follows from the use of model outputs in other studies [41–43]. Locations identified in Quebec and the United States were assigned “high” risk classification using previously established risk measures for the respective areas [44, 45]. Using these location classifications and responses to the question, “How often did you visit woodlands or areas with tall grass and/or shrubs between May and October this year,” we assigned respondents to levels of an “exposure index.” We designated individuals as “negligible” risk when they identified their most frequent activity site in an area with a low likelihood of blacklegged tick presence or if they claimed to visit a high-risk area less than twice during the year. For the remainder of respondents who selected a high-risk area as the site of their most frequent activities in wooded areas, we assigned exposure risk levels of “low” (visited 2 to 10 times during the year), “medium” (11 to 25 times), and “high” (26 visits or more).

#### Statistical modelling

We performed all statistical analyses with R version 4.1.3. We calculated Pearson Chi-square statistics (ɑ=0.05) to test for significant differences between geographic and demographic groups. We used multivariable logistic regression to identify factors associated with high versus low Lyme disease knowledge and high versus low personal adoption of protective practices. First, we performed univariable logistic regression to test for an association of each independent variable with high KS and high PS separately. We retained factors associated with the dependent variable (*p* < 0.2) for each outcome in the initial respective multivariable model [46]. With the MASS package version 7.3.60, we determined final multivariable models for high KS and PS using stepwise selection by Akaike Information Criterion (AIC). We forced gender, age group, education level, and survey region in the final model as potential confounders and exposure index as the primary variable of interest for an association between exposure behaviour and the outcomes. In both models, we excluded individuals who responded “I prefer not to answer” to any questions used as independent variables in statistical analyses. To ensure that our definition of “high” PS was not skewed by scores that lacked responses for property measures which did not apply for some respondents (i.e., use of pesticides and mowing a lawn), we performed a sensitivity analysis of the final multivariable model for high PS that focused on the five individual practices, where the cutoff for a “high” PS in this scenario was 4 measures adopted. We used variance inflation factors, calculated using the mctest package version 1.3.1, to check for multicollinearity in the final models.

#### Spatial analysis

We used ArcGIS Pro 3.1.0 [47] to map the locations respondents identified as their most frequently visited wooded area. We assigned “high” or “low” blacklegged tick probability to each location by executing a spatial join of the activity sites identified by respondents to the data sources used to determine regional Lyme disease risk in Ontario, Quebec, and the United States described above. As most cases of Lyme disease reported in Ottawa disclose that their tick bite occurred during travel outside of the city [5, 31], we explored how far the wooded areas respondents visit most often were from where they live. To determine the distance travelled by each respondent to the site they visited most frequently, we geocoded each respondent to their home postal code. We then calculated the geodesic distance between their home and the activity location they provided based on the latitude and longitude of each location. We used the Summary Statistics tool in ArcGIS to calculate the mean and variance of the distance travelled for all responses and separately for the groups who frequently travelled to locations with a “high” or “low” probability of blacklegged tick presence.

## Results

### Descriptive statistics

A total of 2018 residents of Ottawa participated in this study, with the distribution of respondents roughly even across the five city strata (Table 1). The age group with the greatest proportion of respondents was 55 to 64 (23%, 472), while the fewest respondents were older than 75 (6%, 128). Of the seven family income classes, the highest number of respondents were from families earning $120,000 or more (29%, 515/1777) and of the four education level classes, the highest number of respondents were in the university-educated class (41%, 816/2000). Roughly one-fifth of respondents were Indigenous or other racialized Ottawa residents (Table 1). Overall, 251 respondents (12%) reported having experienced a tick bite, 74 (4%) reported having been personally infected by Lyme disease, and 531 (26%) knew someone who had previously been infected (Table 2). The proportion of respondents who believed Lyme disease to be very serious was 94%. Slightly greater than 40% of respondents reported that they felt at high or medium risk of contracting Lyme disease during the previous spring and summer, yet over half (1,082, 54%) stated that it was an outcome they worry about (Table 2). Approximately one-third did not believe it is easy to protect themselves against infection.

**Table 1:** Descriptive characteristics of survey respondents

	Ottawa, Ontario (2021 Census (%))^3^	n	% (95% CI)
Total	1,017,449 (100)	2018	100
**Region**			
Suburban East	205,776 (18.2)	419	20.8 (18.5, 23.0)
Suburban South	249,938 (22.2)	405	20.1 (17.8, 22.3)
Suburban West	161,625 (14.3)	381	18.9 (16.7, 21.2)
Rural	248,044 (22.0)	380	18.8 (16.6, 21.1)
Urban	262,187 (23.3)	433	21.5 (19.2, 23.7)
**Gender**			
Men	496,045 (48.8)	995	49.3 (47.0, 51.6)
Women	521,405 (51.2)	1015	50.3 (48.0, 52.6)
Other		8	0.4 (0.0, 2.7)
**Age**			
18 to 24	131,170 (12.9) ^3^	139	6.9 (4.8, 9.1)
25 to 34	143,020 (14.1)	242	12.0 (9.9, 14.2)
35 to 44	135,410 (13.3)	303	15.0 (12.9, 17.2)
45 to 54	133,505 (13.1)	343	17.0 (14.9, 19.2)
55 to 64	135,260 (13.3)	472	23.4 (21.3, 25.6)
65 to 74	97,730 (9.6)	391	19.4 (17.2, 21.5)
75 or older	74,415 (7.3)	128	6.3 (4.2, 8.5)
**Population group** ^**1**^			
White	665,960 (65.5)	1592	79.8 (78.1, 81.5)
Indigenous	26,395 (2.6)	55	2.8 (1.1, 4.5)
Other	324,960 (31.9)	349	17.5 (15.8, 19.3)
Total		1996	100.0
**Family income** ^**1,3**^			
< $20,000	17,525 (4.3)	70	3.9 (1.6, 6.3)
$20,000 to $39,999	39,695 (9.7)	148	8.3 (6.0, 10.6)
$40,000 to $59,999	44,940 (11.0)	211	11.9 (9.6, 14.2)
$60,000 to $79,999	49,280 (12.1)	291	16.4 (14.1, 18.7)
$80,000 to $99,999	46,895 (11.5)	298	16.8 (14.5, 19.1)
$100,000 to $119,000	50,875 (12.5)^3^	244	13.7 (11.4, 16.0)
$120,000 or more	158,040 (38.8)^3^	515	29.0 (26.7, 31.3)
Total	407,250	1777	100.0
**Highest education level** ^**1,2,3**^			
High school or less	284,135 (34.1)	296	14.8 (12.5, 17.2)
College	187,210 (22.4)	452	22.6 (20.3, 25.0)
University	225,720 (27.1)	816	40.8 (38.5, 43.2)
Graduate Studies	137,075 (16.4)	436	21.8 (19.5, 24.2)
Total		2000	100.0

^1^Respondents who answered “prefer not to answer” were excluded from totals and proportions

^2^Respondents who answered “other” were excluded from totals and proportions

^3^Ottawa population and demographics figures are Statistics Canada estimates for the Ottawa census subdivision (CSD) from the 2021 census [48]. Region population totals are calculated from Forward Sortation Area (FSA) boundaries which do not fall entirely within the Ottawa CSD; some FSAs classified as rural extend outside of this boundary. Demographics and age group definitions used by Statistics Canada do not directly align with those defined in the survey instrument used in this study (e.g., the survey targeted only adults while the youngest census group spans 15 to 24 years old). Census income groups represent the total number of Ottawa households with the identified income level

**Table 2:** Number and proportion of respondents with knowledge, risk factors, attitudes, and practices regarding Lyme disease. Proportions reported are for column totals

	All responses n (%)	KS ≥ 3 n (%)	KS < 3 n (%)
Total	2018 (100)	1211 (100)	807 (100)
**High Lyme disease knowledge score (KS ≥ 3)**	1211 (60)		
Transmitted by: tick bite	1710 (85)	1185 (98)	525 (65)
Best way to remove tick: pull with tweezers/forceps/other tool	1449 (72)	1109 (92)	340 (42)
First symptom(s): headache/fever/lethargy/reddish rash	1341 (66)	1117 (92)	224 (28)
Detected quickly, Lyme can be treated by: antibiotic tablets	916 (45)	833 (69)	83 (10)
**Lyme disease attitudes** ^**1,3**^			
Feel at high or medium risk	789 (43)	543 (45)	246 (30)
Lyme disease is very serious	1885 (94)	1183 (98)	702 (87)
Easy to protect myself from Lyme disease	1316 (66)	881 (73)	435 (54)
There are great scientific uncertainties about Lyme disease	1081 (54)	706 (58)	375 (46)
Worry about contracting Lyme disease	1082 (54)	713 (59)	369 (46)
**Personal protective measures** ^**2**^			
Seek and remove ticks after stay in forested area^3^	833 (60)	583 (48)	250 (31)
Wearing long clothes (including tucking pants into socks)^3^	1171 (66)	775 (64)	396 (49)
Use insect repellants with DEET or Icaridin^3^	972 (53)	636 (53)	336 (42)
Wear clothing treated with insecticides^3^	222 (13)	117 (10)	105 (13)
Avoid woodlands during spring-to-fall risk period^3^	737 (41)	451 (37)	286 (35)
Use pesticides on my property^3^	153 (9)	77 (6)	86 (11)
Mow lawn regularly on my property^3^	1281 (81)	852 (70)	429 (53)
High personal practices score (PS ≥ 5)	275 (14)	165 (14)	110 (14)
**Exposure risk factors** ^**3**^			
Have access to outdoor yard (not responsible for maintenance)	436 (22)	226 (19)	210 (26)
Have access to outdoor yard (responsible for maintenance)	1315 (66)	883 (73)	432 (54)
Visited woodlands May-October			
More than 25 times	351 (17)	255 (21)	96 (12)
Between 11 and 24 times	312 (16)	205 (17)	107 (13)
Between 1 and 10 times	932 (46)	535 (44)	397 (49)
Never	415 (21)	213 (18)	202 (25)
Frequently travel to high-risk location	1314 (65)	825 (68)	489 (61)
Exposure index (high or medium)	565 (28)	392 (32)	173 (21)
Any travel to high-risk location(s)	1394 (69)	878 (73)	516 (64)
Travel 5 km or less to visit wooded areas May-October	698 (35)	445 (37)	253 (31)
Travel > 10 km to visit wooded areas May-October	541 (27)	350 (29)	191 (24)
Duration of activities in wooded areas typically ≥ 4 h	256 (13)	144 (12)	112 (14)
Walk on trails/cleared paths during activities in wooded areas^2^	1294 (64)	834 (69)	460 (57)
**Primary reason for visits to wooded areas in May-October**			
Work	52 (3)	26 (2)	26 (3)
Recreation	1136 (56)	717 (59)	419 (52)
Birdwatching	153 (8)	84 (7)	69 (9)
Dog walking	337 (17)	205 (17)	132 (16)
Camping	117 (6)	76 (6)	41 (5)
Hunting	19 (1)	9 (1)	10 (1)
Cottage	167 (8)	112 (10)	55 (7)
Own a dog^3^	514 (25)	325 (27)	189 (23)
**Personal history with ticks and Lyme disease** ^**3**^			
Ever had Lyme disease (self-reported)	74 (4)	38 (3)	36 (4)
Diagnosed with Lyme disease by doctor, specialist, naturopath, or private lab testing^4^	23 (1)	12 (1)	11 (1)
Know someone who has had Lyme disease	531 (26)	405 (33)	126 (16)
Ever bitten by a tick	251 (12)	169 (14)	82 (10)

^1^Totals include number of respondents who answered “totally” or “somewhat agree”

^2^Totals include number of respondents who answered “always” or “frequently”

^3^Excludes respondents who answered “Don’t know”, “I prefer not to answer”, or “Does not apply to my situation”

^4^Excludes respondents who declared they have never had Lyme disease

### Lyme disease knowledge

**Fig. 2:**
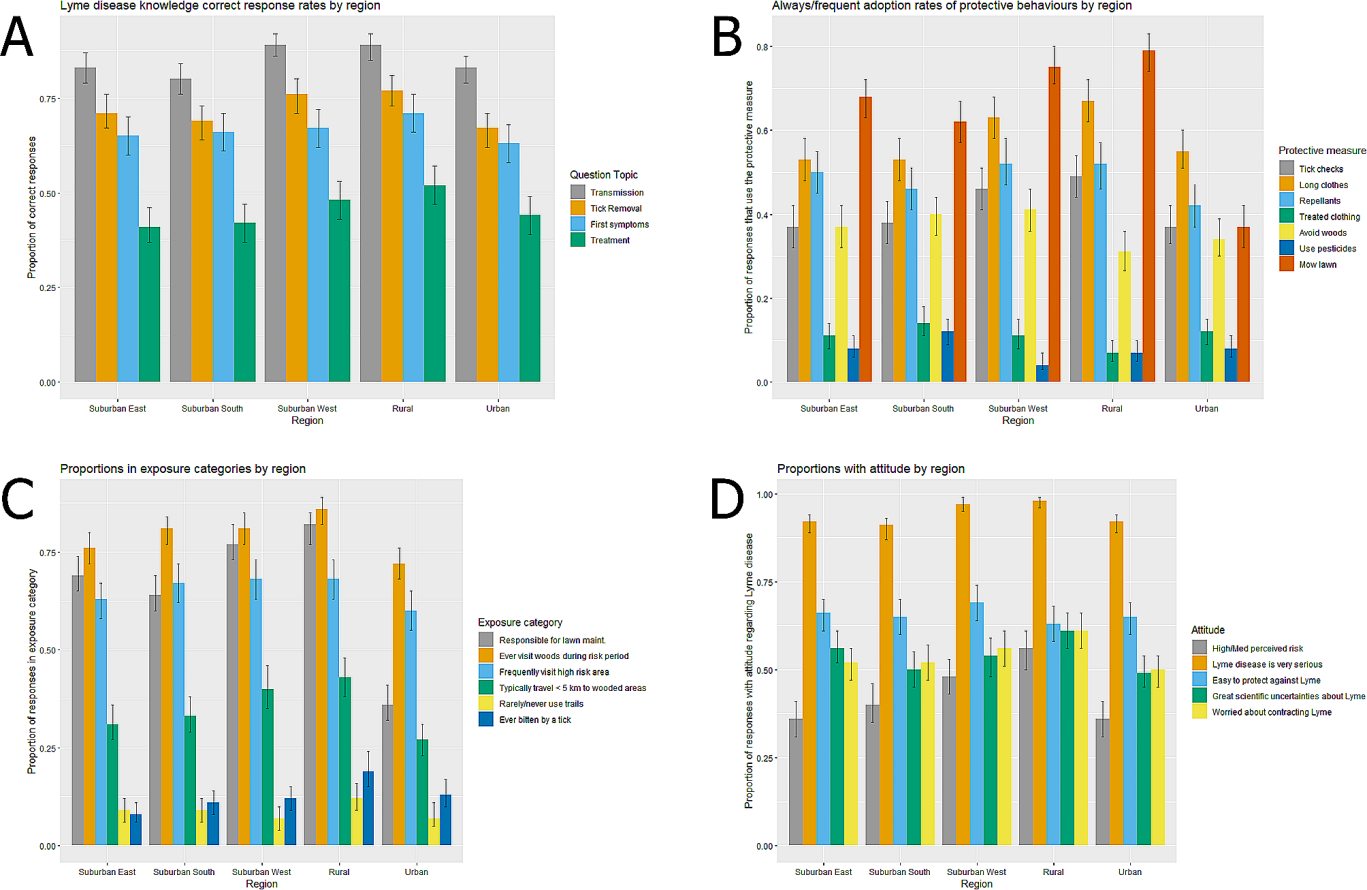
Responses to selected questions by region: (A) Lyme disease knowledge correct response rates, (B) always or frequent adoption rates of protective behaviours, (C) proportions in specified exposure categories, and (D) proportions expressing specified attitudes regarding Lyme disease. Bars represent the proportion of respondents in the respective region who provided the specified answer. Some depicted questions may have different population sizes due to non-applicability

60% of respondents exhibited a high level of Lyme disease knowledge (1,211, Table 2). Respondents aged 55 and older were most likely to demonstrate a high knowledge level (670, 68%) compared to those in the 18 to 34 and 35 to 54 age groups (44% and 58%, respectively, see Supplementary file 3) (*P* < 0.001). Differences in the proportion of subgroups with a high level of Lyme disease knowledge were also statistically significant by gender (*P* = 0.003) and population group (*P* < 0.001). Correct response rates for the knowledge domain questions were consistently highest among respondents from the western suburbs or rural areas, while the proportion of correct answers to each question was generally lowest among urban respondents (Fig. 2A). One exception was knowledge about treatment for Lyme disease, which was the question with the most incorrect responses, regardless of the city survey region. A similar regional pattern was observed in residents’ perceptions of personal risk (Fig. 2D). Of all the knowledge questions, most respondents correctly identified that Lyme disease transmission to humans occurs via tick bites (85%). By comparison, less than half (45%) answered that Lyme could be treated with antibiotic tablets if detected quickly.

### Adoption of personal protective measures

The most common personal protective measures adopted among Ottawa residents were regularly mowing the lawn on one’s property (81%), wearing long clothing (66%), and performing tick checks after stays in wooded areas (60%) (Table 2). The citywide proportion of respondents with a high personal practice score (PS ≥ 5) was 14% and ranged from 11 to 17% across the geographic survey strata (Table 2, Supplementary file 4). 12% of respondents identified no protective measures that they frequently use, while 15% identified a single measure they adopt to protect against Lyme disease. A significantly higher proportion of Indigenous respondents identified practising a high adoption of protective behaviours (24%) compared to other population groups (*P* < 0.05, see Supplementary file 3). Statistically significant differences existed between the proportions of White, Indigenous, and other racialized individuals that perform tick checks (*P* < 0.05), as well as those that wear treated clothing, avoid woodlands between May and October, use pesticides on their property, and mow their lawn (P < < 0.001, see Supplementary file 3). Residents who lived in the western suburbs and rural areas of Ottawa also identified donning long clothing during or performing tick checks after activities in woodland areas more frequently than residents from other city regions (Fig. 2B).

### Exposure risk factors

Several exposure risk factors were common among respondents. Two-thirds of respondents claimed responsibility for maintaining their outdoor yard, and 22% had access to, but no responsibility, for yard space on their residential property. Only 415 respondents (21%) claimed they had never visited a wooded area between May and October of the survey year, with 17% stating that they visited one more than 25 times during the same period (Table 2). Residents living in Ottawa’s urban core identified visiting wooded areas during the risk period less frequently than residents in other city regions (72%, Fig. 2C). Of the primary reasons for visiting wooded areas during the May-October risk period, most respondents did so for recreation (1136, 56%), followed by dog walking (337, 17%).

**Fig. 3:**
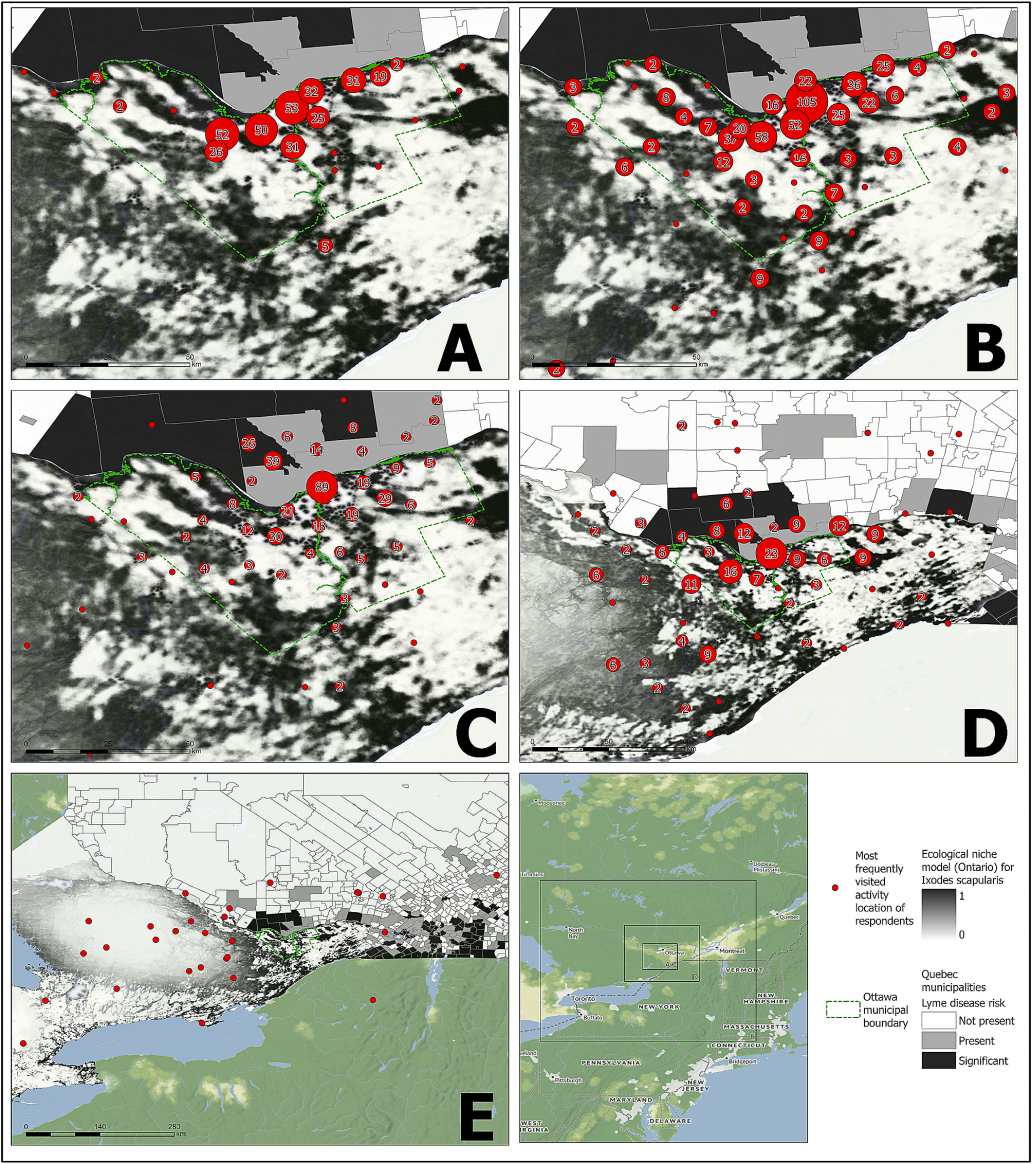
The most frequently visited locations for woodland activities by geodesic distance. Panels group locations by the shortest curvilinear path along the earth’s surface between the respondent’s home and the displayed activity location: (A) less than 2 km, (B) 2 to 10 km, (C) 10 to 30 km, (D) 30 to 125 km, and (E) greater than 125 km. Numbers on maps and point sizes represent the number of respondents who identified locations within that area. The probability of blacklegged tick presence (Ontario) and estimated Lyme disease risk (Quebec) illustrate comparative Lyme disease risk between activity locations. Basemap by Stamen Design, under CC BY 4.0, with data by OpenStreetMap, under ODbL

Figure 3 depicts the sites selected by survey respondents as the woodland site they frequented most often between May and October during the previous year by distance travelled from their home postal code. These maps also display the degree of Lyme disease risk, or the estimated probability of blacklegged tick presence, in the area illustrated by the sources detailed above. In the preceding May to October risk period, 65% of respondents most frequently visited a wooded site that was high-risk for blacklegged tick exposure. Sites in western Ottawa appear to be more frequently visited by residents to whom these sites are local (Fig. 3A) but also by Ottawa residents who travel further within the city to engage in their desired activity (Fig. 3B-D). By comparison, fewer respondents remained in eastern or southern Ottawa or travelled a short distance for local woodland activity. Overall, 35% of respondents claimed to travel less than five kilometres for activities in wooded areas, while the average distance a respondent travelled to a frequently visited site was 18.6 km. 2% of respondents travelled more than 125 km from their home to the wooded location they visit most frequently, predominantly to sites north in the province of Quebec or west of Ottawa in Ontario (Fig. 3E). There was a significant difference (*p* < 0.05) in the average distance travelled between the 82% of respondents (1,314) who visited areas with high Lyme disease risk (15.4 km) versus the remainder (288) who visited areas that represent low risk (33.9 km). High exposure index (entering higher-risk areas more frequently) occurred in a greater proportion of surveyed men compared to women, as well as a smaller proportion of respondents from other racialized groups compared to White and Indigenous individuals (P < < 0.001, see Supplementary file 3).

### Statistical models

In the final Lyme disease knowledge model, the factor most strongly associated with a high knowledge level was residing on a property with personal responsibility for yard maintenance (Odds Ratio (OR) = 2.46, 95% Confidence Interval (CI): 1.75, 3.47; Table 3). Individuals 55 or older also exhibited a significant positive association (OR = 1.92, 95%CI: 1.42, 2.62) compared to respondents in the youngest age group (18 to 34). High Lyme disease knowledge levels were 30% less likely in men than women (OR = 0.69, 95%CI: 0.56, 0.86) and nearly 30% less likely among individuals belonging to non-Indigenous racialized groups compared to White respondents (OR = 0.71, 95%CI: 0.53, 0.95). The likelihood of having a high knowledge level about Lyme disease was 71% greater for respondents with a high exposure index than those with a negligible risk of exposure (OR = 1.71, 95%CI: 1.21, 2.41). Respondents who knew someone else that had been infected by Lyme disease were twice as likely to demonstrate a high level of Lyme disease knowledge (OR = 2.03, 95%CI: 1.57, 2.63). Of all respondents, those who were unable to identify their own perceived risk of Lyme disease (“Don’t Know”) were nearly 80% less likely compared to those who identified their personal risk level as high (OR = 0.23, 95%CI: 0.13, 0.38).

**Table 3:** Factors associated with high knowledge score (KS ≥ 3) regarding Lyme disease (*n* = 1,741)

Factors	Univariable			Multivariable		
	OR	95% CI	P	OR	95% CI	P
**Region (ref: Suburban east)**						
South	0.90	(0.67, 1.21)	0.5	0.90	(0.65, 1.24)	0.5
West	1.07	(0.79, 1.46)	0.7	0.82	(0.59, 1.16)	0.3
Rural	1.33	(0.97, 1.81)	0.08	0.99	(0.70, 1.40)	0.9
Urban	0.83	(0.62, 1.11)	0.2	1.09	(0.79, 1.52)	0.6
**Age (ref: 18 to 34)**						
35 to 54	1.63	(1.24, 2.14)	< 0.001	1.39	(1.02, 1.89)	0.04
55 and older	2.46	(1.09, 3.21)	< 0.001	1.92	(1.42, 2.62)	< 0.001
**Gender (ref: women)**						
Men	0.77	(0.63, 0.93)	0.007	0.69	(0.56, 0.86)	< 0.001
Other	0.61	(0.14, 2.60)	0.5	1.05	(0.21, 5.15)	0.9
**Family income level (ref: $40k to $80k)**						
< $40,000	0.63	(0.45, 0.87)	0.005			
$80,000 +	1.13	(0.91, 1.41)	0.3			
**Education level (ref: High school or less)**						
College	0.99	(0.70, 1.32)	0.8	0.79	(0.55, 1.12)	0.2
University and higher	1.05	(0.79, 1.38)	0.7	1.01	(0.74, 1.39)	0.9
**Population group (ref: White)**						
Indigenous persons	0.51	(0.29, 0.90)	0.02	0.62	(0.33, 1.15)	0.1
Other racialized persons	0.47	(0.36, 0.60)	< 0.001	0.71	(0.53, 0.95)	0.02
**Perceived risk level (ref: high)**						
Medium	1.43	(1.01, 2.03)	0.05	1.35	(0.92, 1.97)	0.1
Low	0.99	(0.71, 1.37)	0.9	0.92	(0.64, 1.32)	0.7
None	0.46	(0.29, 0.73)	< 0.001	0.57	(0.35, 0.95)	0.03
Don’t know/Prefer not to answer	0.19	(0.12, 0.31)	< 0.001	0.23	(0.13, 0.38)	< 0.001
**Outdoor yard (ref: none)**						
Have yard, not responsible for maintenance	1.84	(1.32, 2.58)	< 0.001	1.60	(1.12, 2.30)	0.01
Have yard, responsible for maintenance	3.26	(2.44, 4.39)	< 0.001	2.46	(1.75, 3.47)	< 0.001
**Exposure index (ref: negligible)**						
Low	1.25	(0.97, 1.59)	0.08	1.02	(0.78, 1.34)	0.9
Medium	1.69	(1.26, 2.28)	< 0.001	1.41	(1.01, 1.98)	0.04
High	2.17	(1.61, 2.95)	< 0.001	1.71	(1.21, 2.41)	0.002
**Distance traveled to wooded areas (ref: <1 km)**						
1 to 5 km	0.86	(0.60, 1.22)	0.4			
6 to 10 km	0.66	(0.45, 0.95)	0.03			
11 to 20 km	0.65	(0.44, 0.98)	0.04			
21 + km	1.13	(0.76, 1.65)	0.5			
No travel to wooded areas	0.51	(0.36, 0.72)	< 0.001			
**Duration of woodlands activities (ref: <1 h)**						
1 to 3 h	1.47	(1.14, 1.88)	0.003			
4 to 8 h	0.75	(0.52, 1.08)	0.1			
9 to 24 h	1.52	(0.65, 3.82)	0.3			
> 1 day	1.47	(0.81, 2.79)	0.2			
No travel to wooded areas	0.73	(0.55, 0.98)	0.03			
**Use cleared paths/trails in woodland areas (ref: always)**						
Frequently	1.14	(0.89, 1.47)	0.3			
Rarely	0.79	(0.52, 1.21)	0.3			
Sometimes (not because of Lyme disease)	0.77	(0.51, 1.15)	0.2			
Never	1.01	(0.39, 2.81)	0.9			
No travel to wooded areas	0.63	(0.47. 0.83)	0.001			
Currently own a dog	1.22	(0.98, 1.52)	0.07			
**Ever had Lyme disease (ref: no)**						
Yes	0.65	(0.40, 1.06)	0.09			
Don’t know	1.07	(0.62, 1.86)	0.8			
Know someone who has had Lyme disease	2.65	(2.10, 3.38)	< 0.001	2.03	(1.57, 2.63)	< 0.001
Ever bitten by a tick	1.42	(1.06, 1.92)	0.02			
Primary reason in wooded areas: work	0.63	(0.34, 1.15)	0.1	0.59	(0.31, 1.13)	0.1
Primary reason in wooded areas: fitness/recreation	1.37	(1.13, 1.67)	0.001			
Primary reason in wooded areas: birdwatching	0.73	(0.51, 1.04)	0.08	0.60	(0.40, 0.88)	0.01
Primary reason in wooded areas: dog walking	1.03	(0.80, 1.33)	0.8			
Primary reason in wooded areas: camping	1.19	(0.80, 1.79)	0.4			
Primary reason in wooded areas: hunting	0.49	(0.18, 1.27)	0.1	0.36	(0.12, 1.00)	0.05
Primary reason in wooded areas: cottage	1.35	(0.95, 1.94)	0.1			

Respondents who answered “I prefer not to answer” to any of the explanatory variables were excluded from multivariable analysis

Gender, age, education level, and region were forced into the model as potential confounding variables

Table 4 presents the association between different factors and high personal protective practices scores. In the final model, the factors most strongly associated with high adoption were visiting wooded areas most frequently for work (OR = 2.78, 95%CI: 1.31, 5.67) and having an outdoor yard, with or without responsibility for its maintenance (OR = 2.59, 95%CI: 1.42, 4.98; OR = 2.53, 95%CI: 1.43, 4.78). Respondents who reported previous infection with Lyme disease were more than twice as likely to adopt five or more protective practices (OR = 2.22, 95%CI: 1.19, 4.00). Respondents from non-Indigenous racialized groups were 90% more likely to demonstrate greater adoption than White Ottawa respondents (OR = 1.92, 95%CI: 1.31, 2.79). Respondents who were assigned a high exposure risk index (OR = 0.50, 95%CI: 0.30, 0.80), believed themselves at low risk of infection with Lyme disease (OR = 0.38, 95%CI: 0.25, 0.60), or did not know whether they were at risk (OR = 0.25, 95%CI: 0.11, 0.53) were less likely to adopt protective practices. Results from our sensitivity analysis, wherein property-level measures were removed from the total possible protective practices score, were similar to our primary analysis overall (Supplementary file 5). In this version of the analysis, the major difference was a loss of significance for having a yard and the responsibility for its maintenance. However, having a yard and no maintenance obligations was still associated with more than two times the likelihood (OR = 2.09; 95%CI: 1.27, 3.54; Supplementary file 5) of a respondent demonstrating a high adoption of protective practices compared to having no yard access.

**Table 4:** Factors associated with high Lyme disease protective practices score (PS ≥ 5) (*n* = 1,741)

Factors	Univariable			Multivariable		
	OR	95% CI	P	OR	95% CI	P
**Region (ref: Suburban east)**						
South	1.06	(0.70, 1.61)	0.8	1.01	(0.66, 1.57)	0.9
West	1.02	(0.66, 1.57)	0.9	1.00	(0.64, 1.57)	0.9
Rural	0.89	(0.58, 1.38)	0.6	0.85	(0.53, 1.36)	0.5
Urban	0.66	(0.42, 1.02)	0.06	0.73	(0.45, 1.18)	0.2
**Age (ref: 18 to 34)**						
35 to 54	1.09	(0.74, 1.62)	0.7	1.13	(0.74, 1.75)	0.6
55 and older	0.84	(0.58, 1.24)	0.4	1.04	(0.67, 1.62)	0.9
**Gender (ref: women)**						
Men	1.05	(0.79, 1.38)	0.7	0.98	(0.73, 1.32)	0.9
Other	0.94	(0.05, 5.36)	0.9	0.66	(0.03, 4.62)	0.7
**Family income level (ref: $40k to $80k)**						
< $40,000	0.98	(0.60, 1.57)	0.9			
$80,000 +	1.03	(0.75, 1.42)	0.9			
**Education level (ref: High school or less)**						
College	0.81	(0.52, 1.27)	0.4	0.75	(0.47, 1.21)	0.2
University and higher	0.83	(0.57, 1.23)	0.3	0.72	(0.48, 1.11)	0.1
**Population group (ref: White)**						
Indigenous persons	2.36	(1.16, 4.48)	0.01	1.59	(0.71, 3.31)	0.2
Other racialized persons	1.78	(1.28, 2.47)	< 0.001	1.92	(1.31, 2.79)	< 0.001
**Perceived risk level (ref: high)**						
Medium	0.58	(0.39, 0.87)	0.01	0.62	(0.40, 0.95)	0.03
Low	0.33	(0.22, 0.50)	< 0.001	0.38	(0.25, 0.60)	< 0.001
None	0.50	(0.27, 0.89)	0.02	0.59	(0.30, 1.11)	0.1
Don’t know	0.21	(0.09, 0.44)	< 0.001	0.25	(0.11, 0.53)	< 0.001
**Outdoor yard (ref: none)**						
Have yard, not responsible for maintenance	2.49	(1.41, 4.67)	0.003	2.59	(1.42, 4.98)	0.003
Have yard, responsible for maintenance	2.46	(1.47, 4.44)	0.001	2.53	(1.43, 4.78)	0.003
**Exposure index (ref: negligible)**						
Low	0.91	(0.63, 1.29)	0.6	0.72	(0.49, 1.07)	0.1
Medium	1.02	(0.67, 1.52)	0.9	0.71	(0.45, 1.12)	0.1
High	0.83	(0.53, 1.25)	0.4	0.50	(0.30, 0.80)	0.005
**Distance traveled to wooded areas (ref: <1 km)**						
1 to 5 km	0.94	(0.59, 1.52)	0.8			
6 to 10 km	1.06	(0.65, 1.75)	0.8			
11 to 20 km	1.04	(0.61, 1.79)	0.9			
21 + km	0.71	(0.42, 1.22)	0.2			
No travel to wooded areas	0.57	(0.34, 0.97)	0.04			
**Duration of woodlands activities (ref: <1 h)**						
1 to 3 h	0.83	(0.59, 1.17)	0.3			
4 to 8 h	1.12	(0.68, 1.80)	0.7			
9 to 24 h	0.76	(0.18, 2.28)	0.7			
> 1 day	0.59	(0.20, 1.41)	0.3			
No travel to wooded areas	0.54	(0.34, 0.84)	0.007			
**Use cleared paths/trails in woodland areas (ref: always)**						
Frequently	0.76	(0.54, 1.07)	0.1			
Rarely	1.12	(0.63, 1.91)	0.7			
Sometimes (not because of Lyme disease)	0.77	(0.42, 1.34)	0.4			
Never	0.62	(0.10, 2.26)	0.5			
No travel to wooded areas	0.48	(0.30, 0.74)	0.001			
Currently own a dog	1.25	(0.92, 1.68)	0.1			
**Ever had Lyme disease (ref: no)**						
Yes	3.02	(1.71, 5.13)	< 0.001	2.22	(1.19, 4.00)	0.009
Don’t know	1.51	(0.71, 2.91)	0.3	1.31	(0.60, 2.64)	0.5
Know someone who has had Lyme disease	1.33	(0.98, 1.79)	0.06			
Ever bitten by a tick	1.84	(1.27, 2.62)	0.001	1.62	(1.07, 2.43)	0.02
High Lyme disease knowledge score	0.91	(0.69, 1.20)	0.5			
Primary reason in wooded areas: work	3.49	(1.80, 6.52)	< 0.001	2.78	(1.31, 5.67)	0.006
Primary reason in wooded areas: fitness/recreation	1.38	(1.04, 1.84)	0.03	1.58	(1.14, 2.19)	0.006
Primary reason in wooded areas: birdwatching	1.51	(0.93, 2.37)	0.08			
Primary reason in wooded areas: dog walking	1.05	(0.73, 1.50)	0.8			
Primary reason in wooded areas: camping	1.47	(0.87, 2.39)	0.1			
Primary reason in wooded areas: hunting	1.38	(0.32, 4.28)	0.6			
Primary reason in wooded areas: cottage	1.47	(0.92, 2.27)	0.09	1.80	(1.10, 2.88)	0.02

Respondents who answered “I prefer not to answer” to any of the explanatory variables were excluded from multivariable analysis

Gender, age, education level, and region were forced into the model as potential confounding variables

Multicollinearity was not detected between levels of any independent variables in either the knowledge or the personal protective practices model.

## Discussion

Our survey of residents in a large Canadian municipality sheds light on the level of knowledge and adoption of preventive behaviours concerning Lyme disease in a Canadian region with rapid blacklegged tick emergence. Respondents generally perceived Lyme as a serious disease, and roughly two out of every five individuals believed themselves at high or medium risk during the previous spring and summer. Just over half of respondents worried about contracting Lyme disease regardless of personal risk perception. Our results show that while most Ottawa residents recognized the need to protect themselves against Lyme disease, they were less familiar with the specifics of its transmission, presentation, and treatment. Most surveyed residents identified that the pathogen causing Lyme disease was transmitted exclusively by a bite from an infected tick, consistent with 2017 municipal statistics reported from Rapid Risk Factor Surveillance System surveys conducted by Ottawa Public Health [5]. However, in our results, the overall knowledge demonstrated was low, with 60% of surveyed individuals correctly answering at least three of the four knowledge questions. For population subgroups, a higher knowledge score was significantly more likely among respondents who identified as women, older than 55, or White.

There were notable variations in the level of Lyme disease knowledge among Ottawa residents. Among geographic subgroups, the proportions of respondents who provided the correct answer to each of the knowledge questions were consistently highest in the suburban west and rural regions. Our results demonstrate a similar geographic pattern concerning those who felt at high or medium risk of contracting Lyme disease. Community awareness of the peridomestic presence and abundance of blacklegged ticks in these areas of Ottawa may explain this geographic difference in personal perception of risk and overall Lyme disease knowledge. Prior studies have shown that the highest density of host-seeking ticks [7, 8] and the highest incidence of locally-acquired Lyme disease [31]ence in personal perception of risk and overall Lyme disease knowledge. Prior studies have shown that the highest density of host-seeking ticks [7, 8] and the highest incidence of locally-acquired Lyme disease [31] occur within these same regions of the city. However, our final model of high Lyme disease knowledge did not find a significant association with the city region where the respondent lives, suggesting personal factors might inform Lyme disease knowledge more than location of residence.

Where the threat of tick-borne disease transmission exists, understanding the relationship between population knowledge, exposure potential, and adoption of personal protection is essential to assess educational outreach needs. Three interesting dynamics stood out in our multivariable analyses of composite scores for knowledge and personal practices. First, strong and significant associations existed for the composite scores of knowledge and practices with access to and responsibility for maintaining an outdoor yard. A widely accepted premise in areas where Lyme disease vectors and pathogens are highly endemic, such as the northeast United States, is that a significant proportion of tick exposures resulting in Lyme disease infection occur on residential properties [49]. In contrast, our results indicated that just over 40% of Ottawa residents believe that, locally, it is possible to contract Lyme disease in residential areas. Many Ottawa Lyme disease cases are also attributed to tick bite encounters outside the patient’s home neighbourhood [5, 31]. Even so, Ottawa residents with access to or responsibility for an outdoor yard were more than twice as likely to demonstrate high protective behaviour adoption than those without such outdoor space. Even when property-level protections were removed as a component of the protective practices score in our sensitivity analysis, access to a yard without the need to maintain it retained its significant association with high practices adoption compared to respondents with no yard access. The greater extent of protective measures practiced by residents with access to outdoor yards seems discordant with the perception of residential risk local to Ottawa. This may have multiple causes. One explanation could be that residents with lawns avail themselves of more resources that increase personal awareness of ticks and tick-borne diseases. They may believe their actions consistently reduce or eliminate the backyard risk of tick encounters. Another possibility is that the same respondents believe the local risk of Lyme disease is not high and mow their lawns as a precaution but adopt other measures during recreation as their primary protection method. The adoption rates for individual practices among our sample all fall within the highly variable ranges observed in other studies on preventive and protective practices conducted in Lyme endemic areas [14, 18, 50–52]. Even with a high variability in adoption among the individual measures, our results highlight a clear difference in the likelihood of high knowledge and adoption of protective practices regarding Lyme disease rooted in outdoor yard access. Public health communication may require different strategies for residents without outdoor yards.

Secondly, our study demonstrated significant differences between population sub-groups in the composite scores for knowledge and personal practices adoption. Controlling for other factors, respondents who belonged to a non-Indigenous racialized population group were one-third less likely than White respondents to demonstrate a high knowledge of Lyme disease yet were two-thirds more likely to practise a high adoption of protective behaviours. Despite lower awareness of specific details of Lyme disease transmission and treatment, members of non-Indigenous racialized groups appear to adopt recommended protective behaviours readily. This apparent dissociation between the level of Lyme disease knowledge and personal protection is interesting, as both groups contained a comparable proportion of respondents who believed themselves to be at elevated personal risk (see Supplementary file 3). While a greater proportion of White respondents identified always or frequently performing tick checks after activities in wooded areas, by contrast, nearly half of non-Indigenous racialized respondents declared avoiding woodlands entirely (see Supplementary file 4). Similarly, more than twice the proportion of non-Indigenous racialized respondents indicated a high frequency of using insecticide-treated clothing and pesticides on personal property. Further research into the motivation driving individuals to adopt one or more protective practices, particularly when using one measure at the exclusion of others, could help clarify the dynamics of these personal choices.

We might consider the third dynamic from this analysis as a “false sense of security” gap, characterized by the opposing direction of association with knowledge and personal practices among respondents classified as having a high exposure index. When we adjusted for other factors, these respondents were more likely to demonstrate high Lyme disease knowledge and half as likely to have high levels of adoption of protective practices. We might dismiss this conflicted behaviour as an error in individuals’ risk perception. However, the high exposure index category had the largest proportion of individuals who identified their perceived risk level as high or medium (see Supplementary file 6). It follows that residents who engage in activities contributing to higher exposure risk for Lyme disease do not lack awareness of the local environmental hazard. Instead, these individuals may have been lulled into inaction over time by non-events– that is, they have not previously had a tick encounter or have experienced tick encounters with such relative infrequency that they dismiss additional measures as unnecessary. Another explanation could be that these individuals choose and rely on fewer protective measures at the exclusion of others, with the belief that the actions they take afford them the best balance of safety and personal comfort. Further qualitative research would help identify the processes motivating individuals with high exposure risk to adopt fewer protective measures, including the decisions that lead to adopting or disusing particular measures.

This relationship among respondents with a high exposure index holds particular interest in informing local public health messaging. Our results suggest potential differences in how Ottawa residents translate their perception of risk at home compared to when they engage in outdoor activities elsewhere. In general, a large proportion of respondents located outside the urban core mow private lawns as a form of Lyme disease prevention. Locally, where individuals have responsibility for a property, lawncare and tick exposure risk thus appear to be well-connected in the public consciousness. In contrast, the proportion of respondents who perform tick checks after, or wear long clothing during, activities in woodlands was greater among respondents who live in the city’s western suburbs or rural areas. As noted above [8, 31], these locations represent the municipal region’s most established Lyme disease risk areas. That is, where blacklegged ticks are commonly understood to be abundant, residents’ usage rates of personal protective measures (e.g., tick checks, long clothing) are higher. Yet 65% of survey respondents in our sample frequently visited a site determined to be high-risk based on the probability of established blacklegged tick populations. Moreover, many respondents specified visiting wooded areas in Ottawa’s western Greenbelt– either by crossing the city or merely their backyard. Our models indicate no regional differences exist in either high knowledge or adoption rates of protective practices overall. However, these apparent patterns of travel within the city for activities combined with differences in the adoption of personal protection methods suggest Lyme disease risk may not remain in focus when individuals stray outside their own neighbourhood.

The study design has some limitations which may impact its generalizability. As with any survey, self-selected participation carries the risk that responses may not be broadly representative of the sampled population. Administrators of the survey recruited respondents through a web panel. While they applied recruitment quotas to ensure the sample was representative of national population demographics, the panel selection slightly over-represented older, more educated individuals who may be more likely to enrol and participate. However, internet-administered surveys have provided similar estimates of Lyme disease knowledge and adaptation behaviours compared to other methods [53]. Respondents were asked in the fall to recall their activities and actions up to six months prior and cumulatively over this period. As such, the categorical choices may misrepresent the actual frequency and circumstance of protective behaviours practiced by individuals. We did not ask respondents to identify whether they preferred one measure at the exclusion of all others, which may contribute to an underestimation of associations with strong preventive behaviours [14]. We also can not dismiss the possibility of reverse causality concerning self-perceived risk. As the most practised method of Lyme disease prevention identified in our survey was lawn maintenance, respondents who identified always or frequently applying this measure may believe their likelihood of encountering ticks is low altogether. Given the cross-sectional design, our aim was not to determine the decision processes and conditions driving the selection of specific preventive measures. Prospective research on the use of these methods, whether they are constant or wax and wane throughout a risk season, and the combinations or exclusivity of behaviours, will be necessary to further develop personal and public health strategies - especially in areas where Lyme disease risk is still becoming established. Our results are also partially dependent on our partitioning of Ottawa into the five defined regions. The modifiable areal unit problem of statistical inference should be kept in mind when interpreting any results comparing observations between imposed geographic zones [54–56]. Though we aimed to follow accepted guidelines in the definition of urbanicity, we recognize that these results might differ if we had made different choices in these zones’ definitions. Lastly, we conducted this survey during the COVID-19 pandemic when individuals often had to alter their behaviours. During the period from mid-March until June of 2020, Ottawa Public Health asked residents to limit non-essential outings and businesses remained closed across the province of Ontario. Restrictions enacted by the Ottawa municipal government and National Capital Commission, the agency responsible for federal lands that include Ottawa’s wooded Greenbelt, also included closures of parks for group activities and vehicle access to parking at Greenbelt trailheads. This further limited access to some outdoor spaces from March until July. As a result, people may have visited woodland areas more or less than during the same time in other years, depending on personal circumstances and periodic restrictions that may have been in place.

## Conclusions

The results of this study highlight the interplay between people’s knowledge, patterns of awareness, and levels of vigilance in protection regarding Lyme disease. With insight into these patterns, public health officials might tailor public health messaging to different communities. Timely identification of gaps between the perceived and actual local risk posed by establishing populations of blacklegged ticks is crucial. Outreach that emphasizes the increasing risk in various local outdoor settings– in backyards and beyond– might temper the false sense of security some residents may feel. Enhanced messaging might also present opportunities to emphasize the preventive benefit of adopting multiple complementary protective behaviours. Future analyses should examine the types of activity, locations, and attitudes contributing to reduced personal vigilance in preventing infection with tick-borne pathogens.

### Electronic supplementary material

Below is the link to the electronic supplementary material.

Supplementary Material 1

Supplementary Material 2

Supplementary Material 3

Supplementary Material 4

Supplementary Material 5

Supplementary Material 6

Supplementary Material 7

Supplementary Material 8

## Data Availability

The datasets used and/or analysed during the current study are available from the corresponding author on reasonable request.

## Abbreviations

95% C.I.: 95% Confidence interval
FSA: Forward Sortation Area
KS: Knowledge score
OR: Odds ratio
PMT: Protection Motivation Theory
PS: Personal practices score
