# Supplementary material for: Knowledge, protective behaviours, and perception of Lyme disease in an area of emerging risk: results from a cross-sectional survey of adults in Ottawa, Ontario
Source: BMC Public Health. 2024 Mar 20;24:867. doi: 10.1186/s12889-024-18348-6 (PMC10956326; doi:10.1186/s12889-024-18348-6)
Supplement: Supplementary file 1 — Supplementary Material 1 [file 12889_2024_18348_MOESM1_ESM.pdf]

Stratum	FSA
Urban	K1H
	K1L
	K1M
	K1N
	K1P
	K1R
	K1S
	K1Y
	K1Z
	K2A
	K2B
	K2C
	K2P
Suburban East	K1B
	K1C
	K1E
	K1J
	K1K
	K1W
	K4A
Suburban West	K2H
	K2K
	K2L
	K2M
	K2R
	K2S
	K2T
	K2V
Suburban South	K1T
	K1V
	K2E
	K2G
	K2J
Rural	K0A
	K0G
	K1G
	K1X
	K2W
	K4B
	K4C
	K4M
	K4P
	K7S
