# Supplementary material for: Knowledge, protective behaviours, and perception of Lyme disease in an area of emerging risk: results from a cross-sectional survey of adults in Ottawa, Ontario
Source: BMC Public Health. 2024 Mar 20;24:867. doi: 10.1186/s12889-024-18348-6 (PMC10956326; doi:10.1186/s12889-024-18348-6)
Supplement: Supplementary file 2 — Supplementary Material 2 [file 12889_2024_18348_MOESM2_ESM.pdf]

Survey of Lyme Disease Knowledge, Attitudes and Practices

November 2020 - INSIGHT Research Lab / Leger Opinion

Data Dictionary

Age (LEO)

**0**	Under 18
**1**	Between 18 and 24
**2**	Between 25 and 34
**3**	Between 35 and 44
**4**	Between 45 and 54
**5**	Between 55 and 64
**6**	Between 65 and 74
**7**	75 or older
**9**	I prefer not to answer

Region

**1**	Suburban East
**2**	Suburban South
**3**	Suburban West
**4**	Rural
**5**	Urban
**6**	Other

Q35. What is your gender

**1**	Woman
**2**	Man
**3**	Non-binary/third gender
**96**	Prefer to self-describe
**99**	I prefer not to answer

Q35r96oe. What is your gender – Self description field

Open response question

Q37. Do you identify as an Aboriginal person, that is, First Nations, Métis or Inuk (Inuit)?

**1**	Yes
**2**	No
**3**	I prefer not to answer

Q38. Which population group do you belong to?

**0**	Unchecked
**1**	Checked

Q38r1 White
Q38r2 South Asian (e.g. East Indian, Pakistani, Sri Lankan, etc.)
Q38r3 Chinese
Q38r4 Black
Q38r5 Filipino
Q38r6 Latin American
Q38r7 Arab
Q38r8 Southeast Asian (e.g. Vietnamese, Cambobian, Laotian, Thai, etc.)
Q38r9 West Asian (e.g. Iranian, Afghan, etc.)
Q38r10 Korean
Q38r11 Japanese
Q38r96 Other
Q38r99 I prefer not to answer

Q38r96oe. Which population group do you belong to? – Other

Open response question

Q39. What language do you speak most often at home:

1 English
2 French
3 Both English and French equally
96 Other
99 I prefer not to answer

Q39r96oe. What language do you speak most often at home – Other

Open response question

Q40. What is the total before-tax income of all household members for 2019?

1 $19,999 or less
2 Between $20,000 and $39,999
3 Between $40,000 and $59,999
4 Between $60,000 and $79,999
5 Between $80,000 and $99,999
6 Between $100,000 and $119,999
7 $120,000 or more
9 I prefer not to answer

Q41. What is the highest level of education you have completed?

1 Primary (elementary school)
2 Secondary (high school)
3 College (technical training and/or certificate)
4 University: graduate certificate and/or diploma
5 University: undergraduate degree (Bachelor’s)
6 University: graduate degree (Master’s)

**7**	University: graduate degree (Doctorate)
**96**	Other (specify)
**99**	I prefer not to answer

Q41r96oe. What is the highest level of education you have completed – Other

Open response question

Q1. How long have you lived in Ottawa?

**1**	Less than a year
**2**	1 to 5 years
**3**	6 to 10 years
**4**	More than 10 years
**9**	I prefer not to answer

Q2. Lyme disease is transmitted to humans:

**0**	Unchecked
**1**	Checked

**Q2r1**	By contact with rodents
**Q2r2**	By contact with an individual
**Q2r3**	By contact with an infected dog
**Q2r4**	By a tick bite
**Q2r5**	By a mosquito bite
**Q2r9**	I do not know
**Q2r99**	I prefer not to answer

Q3. The best way to remove a tick attached to your skin is:

**0**	Unchecked
**1**	Checked

**Q3r1**	Pull it out with tweezers/forceps/tick remover/other tool
**Q3r2**	Apply heat, salt or alcohol to the area with the tick
**Q3r3**	Pull/flick/pick it off using your hand
**Q3r96**	Other
**Q3r98**	Don’t know/not sure
**Q3r99**	I prefer not to answer

Q3r96oe. The best way to remove a tick attached to your skin is – Other

Open response question

Q4. The first symptom of Lyme disease is usually:

**0**	Unchecked
**1**	Checked

**Q4r1**	Diarrhea
**Q4r2**	Vomiting
**Q4r3**	Cough
**Q4r4**	Headache
**Q4r5**	Fever
**Q4r6**	A reddish rash on the skin
**Q4r7**	Lethargy/malaise
**Q4r8**	Nasal congestion
**Q4r98**	I do not know
**Q4r99**	I prefer not to answer

Q5. When detected quickly, Lyme disease can be treated:

**0**	Unchecked
**1**	Checked

**Q5r1**	With antibiotics in tablet form
**Q5r2**	With creams applied to the skin
**Q5r3**	With cough medicines
**Q5r4**	There is no treatment for Lyme disease but a preventive vaccine exists
**Q5r5**	There is no treatment or vaccine against Lyme disease
**Q5r8**	I do not know
**Q5r9**	I prefer not to answer

Q6. In Ottawa, it is possible to contract Lyme disease:

**0**	Unchecked
**1**	Checked

**Q6r1**	Only in wooded areas
**Q6r2**	In some wooded areas including public access parks
**Q6r3**	In urban areas
**Q6r4**	In residential areas
**Q6r5**	With current scientific knowledge, it is not known if it is possible to contract Lyme disease in Ottawa
**Q6r6**	It is not possible to contract Lyme disease in Ottawa now
**Q6r8**	I do not know
**Q6r9**	I prefer not to answer

Q7. During the past spring or summer, would you say you are at high risk, medium risk, or no risk of getting Lyme disease?

**1**	High risk
**2**	Medium risk
**3**	Low risk
**4**	No risk
**8**	Don’t know / not sure
**9**	I prefer not to answer

Q8. Lyme disease is a very serious disease:

**1**	Totally agree
**2**	Somewhat agree
**3**	Neither agree or disagree
**4**	Somewhat disagree
**5**	Strongly disagree
**9**	I prefer not to answer

Q9. It would be easy for me to protect myself against Lyme disease:

**1**	Totally agree
**2**	Somewhat agree
**3**	Neither agree or disagree
**4**	Somewhat disagree
**5**	Strongly disagree
**9**	I prefer not to answer

Q10. I have the feeling that there are great scientific uncertainties about Lyme disease:

**1**	Totally agree
**2**	Somewhat agree
**3**	Neither agree or disagree
**4**	Somewhat disagree
**5**	Strongly disagree
**9**	I prefer not to answer

Q11. I am worried about the idea of contracting Lyme disease:

**1**	Totally agree
**2**	Somewhat agree
**3**	Neither agree or disagree
**4**	Somewhat disagree
**5**	Strongly disagree
**9**	I prefer not to answer

Some measures can help prevent Lyme disease. If the following measures were effective AND technically feasible, please choose the options you would consider most acceptable (that it aligns with your personal values and principles). The following measure respects my personal values and principles:

Q12Q20r12. Apply pesticides to the environment to reduce the presence of ticks knowing that non-target species would be killed as well

**1**	Totally acceptable
**2**	Fairly acceptable
**3**	Neither acceptable or unacceptable

4 Fairly unacceptable
5 Totally unacceptable
9 I prefer not to answer

Q12Q20r13. Use biological control techniques in the environment to reduce the presence of ticks (for example, putting fungi into the environment that can reduce ticks)

1 Totally acceptable
2 Fairly acceptable
3 Neither acceptable or unacceptable
4 Fairly unacceptable
5 Totally unacceptable
9 I prefer not to answer

Q12Q20r14. Removing vegetation in forested areas to reduce tick numbers

1 Totally acceptable
2 Fairly acceptable
3 Neither acceptable or unacceptable
4 Fairly unacceptable
5 Totally unacceptable
9 I prefer not to answer

Q12Q20r15. Placing woodchip borders along trails in public access woodlands to reduce tick numbers

1 Totally acceptable
2 Fairly acceptable
3 Neither acceptable or unacceptable
4 Fairly unacceptable
5 Totally unacceptable
9 I prefer not to answer

Q12Q20r16. Protect deer against ticks using feeding stations that apply a topical pesticide to the deer to reduce tick numbers

1 Totally acceptable
2 Fairly acceptable
3 Neither acceptable or unacceptable
4 Fairly unacceptable
5 Totally unacceptable
9 I prefer not to answer

Q12Q20r17. Controlling the number of deer in public access woodlands to reduce the number of ticks

1 Totally acceptable
2 Fairly acceptable
3 Neither acceptable or unacceptable
4 Fairly unacceptable
5 Totally unacceptable

9 I prefer not to answer

Q12Q20r18. Prevent deer from visiting publicly accessible woodlands by putting barriers to reduce the number of ticks

1 Totally acceptable
2 Fairly acceptable
3 Neither acceptable or unacceptable
4 Fairly unacceptable
5 Totally unacceptable
9 I prefer not to answer

Q12Q20r19. Protect small rodents against ticks using baited traps that apply a topical pesticide to reduce the number of infected ticks

1 Totally acceptable
2 Fairly acceptable
3 Neither acceptable or unacceptable
4 Fairly unacceptable
5 Totally unacceptable
9 I prefer not to answer

Q12Q20r20. Vaccinate small rodents to protect them against the bacteria that causes Lyme disease to reduce the number of infected ticks

1 Totally acceptable
2 Fairly acceptable
3 Neither acceptable or unacceptable
4 Fairly unacceptable
5 Totally unacceptable
9 I prefer not to answer

Please identify the measures you currently use to protect yourself from Lyme disease. In Ottawa, I apply this measure to protect myself from Lyme disease:

Q21Q27r21. Seeking and removing ticks on yourself after a stay in a forested area

1 Always
2 Frequently
3 Rarely
4 Never
5 Sometimes I apply this measure, but not to protect myself from Lyme disease
6 Does not apply to my situation
9 I prefer not to answer

Q21Q27r22. Wearing long clothes that covers the legs (for example, tuck pants into socks)

1 Always
2 Frequently
3 Rarely
4 Never
5 Sometimes I apply this measure, but not to protect myself from Lyme disease
6 Does not apply to my situation
9 I prefer not to answer

Q21Q27r23. Use insect repellants with DEET or Icaridin on skin and / or clothing

1 Always
2 Frequently
3 Rarely
4 Never
5 Sometimes I apply this measure, but not to protect myself from Lyme disease
6 Does not apply to my situation
9 I prefer not to answer

Q21Q27r24. Wear clothing treated with an insecticide (e.g. permethrin or InsectShield)

1 Always
2 Frequently
3 Rarely
4 Never
5 Sometimes I apply this measure, but not to protect myself from Lyme disease
6 Does not apply to my situation
9 I prefer not to answer

Q21Q27r25. Avoid woodlands during the spring-to-fall risk period

1 Always
2 Frequently
3 Rarely
4 Never
5 Sometimes I apply this measure, but not to protect myself from Lyme disease
6 Does not apply to my situation
9 I prefer not to answer

Q21Q27r26. Put pesticides on my property

1 Always
2 Frequently
3 Rarely
4 Never
5 Sometimes I apply this measure, but not to protect myself from Lyme disease

6 Does not apply to my situation
9 I prefer not to answer

Q21Q27r27. Mow the lawn regularly on my property

- **1** Always
- **2** Frequently
- **3** Rarely
- **4** Never
- **5** Sometimes I apply this measure, but not to protect myself from Lyme disease
- **6** Does not apply to my situation
- **9** I prefer not to answer

Q28. With regard to the outside environment of your personal home, what is the statement that best applies to your situation

- **1** I do not have access to an outdoor yard
- **2** I have access to an outdoor yard but I do not have the responsibility for its maintenance
- **3** I have access to an outdoor yard and I have the responsibility for its maintenance
- **9** I prefer not to answer

Q29. How often did you visit woodlands or areas with tall grass and/or shrubs between May and October this year:

- **1** More than 25 times
- **2** From 11 to 25 times
- **3** From 2 to 10 times
- **4** Less than 2 times
- **5** Never
- **9** I prefer not to answer

Q29a. Thinking of the wooded areas or areas with tall grass and/or shrubs that you visited between May and October, please select the location you visited most frequently by clicking on the map below. You may click on the map until the pin appears in the correct location. When you are satisfied with the location of the pin, click 'Continue'. You will be able to select up to two additional locations in the following questions.

Latitude/Longitude; GCS decimal degrees

Q29a2NA. If you visited other wooded areas or areas with tall grass and/or shrubs between May and October, please select the location you visited by clicking on the map below. If you did not visit additional areas click on "No others". – NO OTHERS

- **0** Unchecked
- **1** Checked

Q29a2. If you visited other wooded areas or areas with tall grass and/or shrubs between May and October, please select the location you visited by clicking on the map below. If you did not visit additional areas click on "No others".

Latitude/Longitude; GCS decimal degrees

Q29a3NA. Any others? – NO OTHERS

**0**	Unchecked
**1**	Checked

Q29a3. Any others?

Latitude/Longitude; GCS decimal degrees

Q29b. Still thinking of the wooded areas or areas with tall grass and/or shrubs that you visited between May and October, how far from your home did you typically travel?

**1**	< 1 kilometre
**2**	1 to 5 kilometres
**3**	6 to 10 kilometres
**4**	11 to 20 kilometres
**5**	21+ kilometres
**9**	I prefer not to answer

Q29c. Still thinking of the wooded areas or areas with tall grass and/or shrubs that you visited between May and October, how long do you typically spend during these activities?

**1**	< 1 hour
**2**	1 to 3 hours
**3**	4 to 8 hours
**4**	9 to 24 hours
**5**	> 1 day
**9**	I prefer not to answer

Q29d. When you visited the wooded areas or areas with tall grass and/or shrubs between May and October, how often did you walk on cleared paths and trails, avoiding tall grass and plants?

**1**	Always
**2**	Frequently
**3**	Rarely
**4**	Sometimes, but not because of Lyme disease
**5**	Never
**6**	Not applicable
**9**	I prefer not to answer

Q29e. What were the primary reasons for your visits to wooded areas or areas with tall grass and/or shrubs between May and October?

Q29er1. For work

0 Unchecked
1 Checked

Q29er2. For fitness or recreational activities (hiking/running/biking, etc.)

0 Unchecked
1 Checked

Q29er3. For birdwatching

0 Unchecked
1 Checked

Q29er4. For dog walking

0 Unchecked
1 Checked

Q29er5. For camping

0 Unchecked
1 Checked

Q29er6. For hunting

0 Unchecked
1 Checked

Q29er7. For going to a cottage

0 Unchecked
1 Checked

Q29er96. Other

0 Unchecked
1 Checked

Q29er99. I prefer not to answer

0 Unchecked
1 Checked

Q29er96oe. What were the primary reasons for your visits to wooded areas or areas with tall grass and/or shrubs between May and October – Other

Open response question

Q30. Do you have a dog currently?

1 Yes
2 No
3 I prefer not to answer

Q31. Have you ever had Lyme disease?

- **1** Yes
- **2** No
- **8** Don’t know
- **9** I prefer not to answer

Q31a. Were you diagnosed with Lyme disease by a family physician, general practitioner or medical specialist (e.g. infectious disease specialist, neurologist, cardiologist)?

- **1** Yes
- **2** No
- **3** I prefer not to answer

Q31b. Were you diagnosed with Lyme disease by a naturopath or by other means (e.g. private laboratory testing)?

- **1** Yes
- **2** No
- **3** I prefer not to answer

Q32. Do you personally know anyone who has ever had Lyme disease?

- **1** Yes
- **2** No
- **3** I prefer not to answer

Q33. To your knowledge, have you ever been bitten by a tick?

- **1** Yes
- **2** No
- **3** I prefer not to answer
