# Supplementary material for: Knowledge, protective behaviours, and perception of Lyme disease in an area of emerging risk: results from a cross-sectional survey of adults in Ottawa, Ontario
Source: BMC Public Health. 2024 Mar 20;24:867. doi: 10.1186/s12889-024-18348-6 (PMC10956326; doi:10.1186/s12889-024-18348-6)
Supplement: Supplementary file 3 — Supplementary Material 3 [file 12889_2024_18348_MOESM3_ESM.docx]

**Supplementary Table 1. Proportions of Lyme disease knowledge, risk factors, attitudes and practices by select sample subgroups**

|  | Gender  n (%) | | | Age group  n (%) | | | Population Group^1^ n (%) | | | Total  % |
| --- | --- | --- | --- | --- | --- | --- | --- | --- | --- | --- |
|  | Men | Women | Other | 18 to 34 | 35 to 54 | 55+ | White | Indigenous | Other |  |
| Total | 995 | 1015 | 8 | 381 | 646 | 991 | 1592 | 55 | 349 | 2018 |
| High Lyme disease knowledge score (KS ≥ 3) | 562 (56) | 645 (64)^*^ | 4 (50) | 166 (44)^**^ | 375 (58) | 670 (68)^**^ | 1018 (64)^**^ | 25 (45) | 154 (44) | 1211 (60) |
| Perceived risk (high or medium)^1^ | 387 (42) | 399 (44) | 3 (43) | 145 (44) | 280 (48)^*^ | 364 (39)^*^ | 618 (42) | 32 (64)^*^ | 132 (44) | 789 (43) |
| High personal practices score (PS ≥ 5) | 134 (13) | 140 (14) | 1 (13) | 53 (14) | 98 (15) | 124 (13) | 194 (12)^*^ | 13 (24)^*^ | 60 (17) | 275 (14) |
| Seek and remove ticks^1^ | 415 (57) | 416 (64)^*^ | 2 (33)^*^ | 157 (58) | 281 (60) | 395 (61) | 685 (61) | 28 (70)^*^ | 109 (51)^*^ | 833 (60) |
| Wear long clothing^1^ | 560 (63)^*^ | 606 (69) | 5 (71) | 206 (61) | 369 (63) | 596 (70)^*^ | 925 (66) | 32 (68) | 200 (67) | 1171 (66) |
| Use repellents^1^ | 470 (52) | 499 (54) | 3 (43) | 164 (48) | 332 (56) | 476 (53) | 779 (53) | 27 (55) | 159 (52) | 972 (53) |
| Wear treated clothing^1^ | 121 (14) | 101 (12) | 0 (0) | 56 (17)^*^ | 77 (14) | 89 (11)^*^ | 139 (10) | 11 (23)^**^ | 68 (24)^**^ | 222 (13) |
| Avoid woodlands^1^ | 337 (38) | 397 (45)^*^ | 3 (43) | 126 (38) | 231 (39) | 380 (45)^*^ | 547 (39) | 24 (50)^**^ | 157 (51)^**^ | 737 (41) |
| Use pesticides^1^ | 98 (12) | 65 (8)^*^ | 0 (0)^*^ | 45 (16)^**^ | 67 (13) | 51 (6)^**^ | 97 (8) | 12 (29)^**^ | 49 (18)^**^ | 163 (10) |
| Mow lawn^1^ | 640 (79) | 637 (84)^*^ | 4 (67)^*^ | 151 (59)^**^ | 443 (83) | 687 (87) | 1061 (84) | 28 (68)^**^ | 180 (71)^**^ | 1281 (81) |
| Exposure index (high or medium) | 322 (32)^**^ | 242 (24) | 1 (14)^**^ | 104 (27) | 198 (31) | 263 (27) | 480 (30) | 17 (31) | 62 (18)^**^ | 565 (28) |
| Walk on trails/paths during activities in wooded areas^1^ | 660 (67) | 628 (62) | 6 (75) | 227 (60) | 446 (69)^*^ | 621 (63) | 1063 (67) | 36 (65) | 185 (53)^**^ | 1294 (64) |
| Own a dog^1^ | 277 (28) | 234 (23) | 3 (38)^*^ | 105 (28)^*^ | 184 (28)^*^ | 225 (23) | 425 (27) | 30 (55)^**^ | 58 (17)^**^ | 514 (25) |
| Had Lyme disease^1^ | 43 (4) | 31 (3) | 0 (0) | 22 (6) | 28 (5) | 24 (2)^*^ | 49 (3) | 8 (15)^**^ | 16 (5)^**^ | 74 (4) |
| Know someone who has had Lyme disease | 229 (23) | 299 (29)^**^ | 3 (38)^**^ | 70 (19)^**^ | 156 (24) | 305 (31)^**^ | 456 (29) | 18 (33)^**^ | 53 (15)^**^ | 531 (26) |
| Ever bitten by a tick | 153 (15)^**^ | 95 (9) | 3 (38)^**^ | 39 (2) | 82 (13) | 130 (13) | 203 (13) | 15 (27)^**^ | 26 (8)^**^ | 251 (12) |

*^1^ Respondents who answered “Don’t know”, “I prefer not to answer”, or “Does not apply to my situation” were excluded.
^*^ Significant (p < 0.05) difference when compared to other genders, age, or population groups (Pearson Chi-square statistic, or Fisher’s exact test where appropriate)
^**^ Significant (p < 0.001) difference when compared to other genders, age, or population groups (Pearson Chi-square statistic, or Fisher’s exact test where appropriate)*
