# Supplementary material for: Knowledge, protective behaviours, and perception of Lyme disease in an area of emerging risk: results from a cross-sectional survey of adults in Ottawa, Ontario
Source: BMC Public Health. 2024 Mar 20;24:867. doi: 10.1186/s12889-024-18348-6 (PMC10956326; doi:10.1186/s12889-024-18348-6)
Supplement: Supplementary file 4 — Supplementary Material 4 [file 12889_2024_18348_MOESM4_ESM.docx]

**Supplementary Table 2. Personal protective measure responses by population group**

|  | White^1^ | | | | | Indigenous persons^1^ | | | | | Other racialized persons^1^ | | | | |
| --- | --- | --- | --- | --- | --- | --- | --- | --- | --- | --- | --- | --- | --- | --- | --- |
|  | Always | Frequently | Rarely | Never / Does not apply | Sometimes^*^ | Always | Frequently | Rarely | Never / Does not apply | Sometimes^*^ | Always | Frequently | Rarely | Never / Does not apply | Sometimes^*^ |
| Total  (%) | n = 1592 | | | | | n = 55 | | | | | n = 349 | | | | |
| Seek and remove ticks after a stay in a forested area | 360  (23) | 325  (20) | 252  (16) | 602  (38) | 45  (3) | 16  (29) | 12  (22) | 6  (11) | 17  (31) | 3  (5) | 57  (16) | 52  (15) | 51  (15) | 169  (48) | 15  (4) |
| Wear long clothing that covers the legs | 390  (24) | 535  (34) | 269  (17) | 294  (18) | 98  (6) | 14  (25) | 18  (33) | 7  (13) | 11  (20) | 5  (9) | 92  (26) | 108  (31) | 51  (15) | 70  (20) | 25  (7) |
| Use insect repellants with DEET or Icaridin | 289  (18) | 490  (31) | 359  (23) | 306  (19) | 142  (9) | 14  (25) | 13  (24) | 10  (18) | 10  (18) | 7  (13) | 71  (20) | 88  (25) | 63  (18) | 86  (25) | 38  (11) |
| Wear clothing treated with insecticide | 55  (3) | 84  (5) | 196  (12) | 1222  (77) | 27  (2) | 6  (11) | 5  (9) | 8  (15) | 32  (58) | 4  (7) | 30  (9) | 38  (11) | 44  (13) | 223  (64) | 10  (3) |
| Avoid woodlands during the spring-to-fall risk period | 150  (9) | 397  (25) | 422  (27) | 537  (34) | 78  (5) | 10  (18) | 14  (25) | 12  (22) | 13  (24) | 6  (11) | 60  (17) | 97  (28) | 76  (22) | 84  (24) | 27  (8) |
| Put pesticides on my property | 33  (2) | 64  (4) | 175  (11) | 959  (60) | 57  (4) | 5  (9) | 7  (13) | 5  (9) | 34  (62) | 3  (5) | 21  (6) | 28  (8) | 56  (16) | 227  (65) | 13  (4) |
| Mow the lawn regularly on my property | 681  (43) | 380  (24) | 57  (4) | 381  (24) | 85  (5) | 19  (35) | 9  (16) | 7  (13) | 15  (27) | 3  (5) | 83  (24) | 97  (28) | 31  (9) | 109  (31) | 23  (7) |

^*^ Sometimes indicates “Sometimes I apply this measure, but not to protect myself from Lyme disease”.
^1^ “I prefer not to answer” responses are not presented under population group responses.
