# Supplementary material for: Knowledge, protective behaviours, and perception of Lyme disease in an area of emerging risk: results from a cross-sectional survey of adults in Ottawa, Ontario
Source: BMC Public Health. 2024 Mar 20;24:867. doi: 10.1186/s12889-024-18348-6 (PMC10956326; doi:10.1186/s12889-024-18348-6)
Supplement: Supplementary file 5 — Supplementary Material 5 [file 12889_2024_18348_MOESM5_ESM.docx]

**Supplementary Table 3. Results from sensitivity analysis for factors associated with high Lyme disease protective practices score (PS ≥ 4) on a scale of 5 total measures (n = 1,741)**

| **Factors** | **Multivariable** | | |
| --- | --- | --- | --- |
|  | **OR** | **95% CI** | ***P*** |
| Region (ref: Suburban east) |  |  |  |
| South | 0.89 | (0.58, 1.38) | 0.6 |
| West | 1.10 | (0.71, 1.70) | 0.7 |
| Rural | 0.82 | (0.51, 1.29) | 0.4 |
| Urban | 0.91 | (0.58, 1.40) | 0.7 |
| Age (ref: 18 to 34) |  |  |  |
| 35 to 54 | 1.33 | (0.88, 2.03) | 0.2 |
| 55 and older | 1.25 | (0.83, 1.92) | 0.3 |
| Gender (ref: women) |  |  |  |
| Men | 0.95 | (0.71, 1.26) | 0.7 |
| Other | 0.69 | (0.03, 4.55) | 0.7 |
| Education level (ref: High school or less) |  |  |  |
| College | 0.76 | (0.49, 1.20) | 0.2 |
| University and higher | 0.69 | (0.47, 1.04) | 0.07 |
| Population group (ref: white) |  |  |  |
| Indigenous persons | 1.39 | (0.63, 2.87) | 0.4 |
| Other racialized persons | 1.87 | (1.30, 2.68) | <0.001 |
| Perceived risk level (ref: high) |  |  |  |
| Medium | 0.58 | (0.38, 0.88) | 0.01 |
| Low | 0.35 | (0.23, 0.53) | <0.001 |
| None | 0.48 | (0.25, 0.89) | 0.02 |
| Don’t know | 0.34 | (0.17, 0.65) | 0.002 |
| Outdoor yard (ref: none) |  |  |  |
| Have yard, not responsible for maintenance | 2.09 | (1.27, 3.54) | 0.005 |
| Have yard, responsible for maintenance | 1.34 | (0.82, 2.24) | 0.3 |
| Exposure index (ref: negligible) |  |  |  |
| Low | 0.91 | (0.63, 1.32) | 0.6 |
| Medium | 0.79 | (0.50, 1.21) | 0.3 |
| High | 0.54 | (0.33, 0.85) | 0.01 |
| Ever had Lyme disease (ref: no) |  |  |  |
| Yes | 2.37 | (1.30, 4.19) | 0.004 |
| Don’t know | 1.52 | (0.74, 2.93) | 0.2 |
| Know someone who has had Lyme disease | 1.38 | (1.01, 1.87) | 0.04 |
| Ever bitten by a tick |  |  |  |
| Primary reason in wooded areas: work | 2.52 | (1.19, 5.11) | 0.01 |
| Primary reason in wooded areas: fitness/recreation | 1.53 | (1.12, 2.10) | 0.007 |
| Primary reason in wooded areas: cottage | 1.60 | (0.98, 2.54) | 0.05 |

Respondents who answered “I prefer not to answer” to any of the explanatory variables were excluded from multivariable analysis.
Gender, age, education level, and region were forced into the model as potential confounding variables.
