# Supplementary material for: Knowledge, protective behaviours, and perception of Lyme disease in an area of emerging risk: results from a cross-sectional survey of adults in Ottawa, Ontario
Source: BMC Public Health. 2024 Mar 20;24:867. doi: 10.1186/s12889-024-18348-6 (PMC10956326; doi:10.1186/s12889-024-18348-6)
Supplement: Supplementary file 6 — Supplementary Material 6 [file 12889_2024_18348_MOESM6_ESM.docx]

**Supplementary Table 4. Ottawa city region, risk perception, and past tick bite status by exposure index level**

|  |  |  | | Exposure Index | | | |
| --- | --- | --- | --- | --- | --- | --- | --- |
|  |  | Total | Negligible | | Low | Medium | High |
| Total  n (%) |  | 2018 | 1018 | | 435 | 267 | 298 |
| Region^1^ | Suburban East | 419 | 233  (56) ^**^ | | 82  (20) | 58  (14) | 46  (11) |
|  | Suburban South | 405 | 203  (50) | | 91  (22) | 57  (14) | 54  (13) |
|  | Suburban West | 381 | 177  (46) | | 85  (22) | 38  (10) | 81  (21) ^**^ |
|  | Rural | 380 | 162  (43) | | 92  (24) | 51  (13) | 75  (20)^**^ |
|  | Urban | 433 | 243  (56) ^**^ | | 85  (20) | 63  (15) | 42  (10) |
| Perceived risk^1,2^ | None | 155 | 130  (84) ^**^ | | 19  (12) ^**^ | 6  (4) | 0  (0) |
|  | Low | 893 | 454  (51) | | 218  (24) | 122  (14) | 99  (11) |
|  | Medium | 576 | 205  (36) | | 136  (24) | 106  (18) | 129  (22) ^**^ |
|  | High | 213 | 93  (44) | | 42  (20) | 26  (12) | 52  (24) ^**^ |
| Ever bitten by a tick^1,2^ | Yes | 251 | 71  (28) | | 57  (23) | 47  (19) | 76  (30) ^**^ |
|  | No | 1763 | 945  (54) ^**^ | | 377  (21) | 219  (12) | 222  (13) |

^1^ *Proportions are calculated for row totals.*^2^ *Respondents who answered “Don’t know / not sure” or “I prefer not to answer” were excluded.
^*^ Significant (p < 0.05) difference when compared to other genders, age, or population groups (Pearson Chi-square statistic, or Fisher’s exact test where appropriate)
^**^ Significant (p < 0.001) difference when compared to other genders, age, or population groups (Pearson Chi-square statistic, or Fisher’s exact test where appropriate)*
