# Supplementary material for: Knowledge, protective behaviours, and perception of Lyme disease in an area of emerging risk: results from a cross-sectional survey of adults in Ottawa, Ontario
Source: BMC Public Health. 2024 Mar 20;24:867. doi: 10.1186/s12889-024-18348-6 (PMC10956326; doi:10.1186/s12889-024-18348-6)
Supplement: Supplementary file 7 — Supplementary Material 7 [file 12889_2024_18348_MOESM7_ESM.docx]

**Supplementary Table 5. Knowledge and personal practice measure scores^1^ by population groups and regions**

|  |  | Knowledge score | | | | | Personal practice score | | | | | | | |
| --- | --- | --- | --- | --- | --- | --- | --- | --- | --- | --- | --- | --- | --- | --- |
|  | Total  n (%) | 0 | 1 | 2 | 3 | 4 | 0 | 1 | 2 | 3 | 4 | 5 | 6 | 7 |
| White | 1592 | 45 (3) | 173 (11) | 356 (22) | 489 (31) | 529 (33) | 164(10) | 244 (15) | 329 (21) | 367 (23) | 294 (18) | 129 (8) | 46 (3) | 19 (1) |
| Indigenous persons | 55 | 8 (15) | 13 (24) | 9 (16) | 12 (22) | 13 (24) | 11 (20) | 6 (11) | 5 (9) | 12 (22) | 8 (15) | 6 (11) | 1 (2) | 6 (11) |
| Other racialized persons | 349 | 43 (12) | 59 (17) | 93 (27) | 94 (27) | 60 (17) | 66 (19) | 46 (13) | 70 (20) | 53 (15) | 54 (15) | 21 (6) | 17 (5) | 22 (6) |
| Suburban East | 419 | 21 (5) | 49 (12) | 99 (24) | 153 (37) | 97 (23) | 54 (13) | 69 (16) | 79 (19) | 86 (21) | 71 (17) | 34 (8) | 15 (4) | 11 (3) |
| Suburban South | 405 | 25 (6) | 57 (14) | 95 (23) | 117 (29) | 111 (27) | 60 (15) | 55 (14) | 80 (20) | 84 (21) | 66 (16) | 33 (8) | 15 (4) | 12 (3) |
| Suburban West | 381 | 14 (4) | 37 (10) | 91 (24) | 108 (28) | 131 (34) | 27 (7) | 46 (12) | 80 (21) | 91 (24) | 74 (19) | 42 (11) | 14 (4) | 7 (2) |
| Rural | 380 | 12 (3) | 34 (9) | 79 (21) | 113 (30) | 142 (37) | 24 (6) | 45 (12) | 77 (20) | 94 (25) | 94 (25) | 31 (8) | 10 (3) | 5 (1) |
| Urban | 433 | 25 (6) | 71 (16) | 98 (23) | 109 (25) | 130 (30) | 81 (19) | 85 (20) | 90 (21) | 78 (18) | 53 (12) | 21 (5) | 13 (3) | 12 (3) |

^1^ Scores represent the number of protective measures respondents indicated they use “Always” or “Frequently”.
