# Supplementary material for: Knowledge, protective behaviours, and perception of Lyme disease in an area of emerging risk: results from a cross-sectional survey of adults in Ottawa, Ontario
Source: BMC Public Health. 2024 Mar 20;24:867. doi: 10.1186/s12889-024-18348-6 (PMC10956326; doi:10.1186/s12889-024-18348-6)
Supplement: Supplementary file 8 — Supplementary Material 8 [file 12889_2024_18348_MOESM8_ESM.docx]

#### Supplementary Materials

Supplementary file 1

PDF (.pdf)

Forward Sortation Areas (FSAs) included in each survey sub-region

Provides a list of all FSAs that defined survey sub-regions across the City of Ottawa, to geographically stratify respondents within the municipal region

Supplementary file 2

PDF (.pdf)

Lyme disease knowledge, attitudes, and practices questionnaire

Questionnaire used to design the web survey administered to participants

Supplementary file 3

DOC (.docx)

Proportions of Lyme disease knowledge, risk factors, attitudes and practices by select sample subgroups

A summary of selected survey topics with answers stratified by gender, age group, and population group.

Supplementary file 4

DOC (.docx)

Personal protective measure responses by population group

A summary of personal protective measure responses identifying the frequency of use for each measure (always, frequently, rarely, never/does not apply, sometimes), stratified by population group.

Supplementary file 5

DOC (.docx)

Results from sensitivity analysis for factors associated with high Lyme disease protective practices score (PS = 4 or 5) from a total of 5 personal measures.

Presents the results of the final multivariable model when a PS of 4 or 5 is considered high adoption of practices after removing property-level protections from the maximum total PS.

Supplementary file 6

DOC (.docx)

Ottawa city region, risk perception, and past tick bite status by exposure index level

Presents the proportional breakdown of exposure index levels (negligible, low, medium, high) across (1) geographic strata, (2) self-identified perception of Lyme disease risk, and (3) self-reported history of a tick bite.

Supplementary file 7

DOC (.docx)

Knowledge and personal practice measure scores by population groups and regions

Identifies the proportion within each population group and geographic strata that demonstrated the given composite score for knowledge (KS) and protective practices (PS).
